# Cytotoxicity, metabolism, and isozyme mapping of the synthetic cannabinoids JWH-200, A-796260, and 5F-EMB-PINACA studied by means of in vitro systems

**DOI:** 10.1007/s00204-021-03148-3

**Published:** 2021-08-28

**Authors:** Tanja M. Gampfer, Lea Wagmann, Anouar Belkacemi, Veit Flockerzi, Markus R. Meyer

**Affiliations:** 1 Department of Experimental and Clinical Toxicology, Institute of Experimental and Clinical Pharmacology and Toxicology, Center for Molecular Signaling (PZMS), Saarland University, 66421 Homburg, Germany; 2 Department of Experimental and Clinical Pharmacology, Institute of Experimental and Clinical Pharmacology and Toxicology, Center for Molecular Signaling (PZMS), Saarland University, 66421 Homburg, Germany

**Keywords:** Synthetic cannabinoids, Cytotoxicity, HepG2, Imaging, Isozyme mapping, Metabolism

## Abstract

Intake of synthetic cannabinoids (SC), one of the largest classes of new psychoactive substances, was reported to be associated with acute liver damage but information about their hepatotoxic potential is limited. The current study aimed to analyze the hepatotoxicity including the metabolism-related impact of JWH-200, A-796260, and 5F-EMB-PINACA in HepG2 cells allowing a tentative assessment of different SC subclasses. A formerly adopted high-content screening assay (HCSA) was optimized using a fully automated epifluorescence microscope. Metabolism-mediated effects in the HCSA were additionally investigated using the broad CYP inhibitor 1-aminobenzotriazole. Furthermore, phase I metabolites and isozymes involved were identified by in vitro assays and liquid chromatography–high-resolution tandem mass spectrometry. A strong cytotoxic potential was observed for the naphthoylindole SC JWH-200 and the tetramethylcyclopropanoylindole compound A-796260, whereas the indazole carboxamide SC 5F-EMB-PINACA showed moderate effects. Numerous metabolites, which can serve as analytical targets in urine screening procedures, were identified in pooled human liver microsomes. Most abundant metabolites of JWH-200 were formed by *N*-dealkylation, oxidative morpholine cleavage, and oxidative morpholine opening. In case of A-796260, most abundant metabolites included an oxidative morpholine cleavage, oxidative morpholine opening, hydroxylation, and dihydroxylation followed by dehydrogenation. Most abundant 5F-EMB-PINACA metabolites were generated by ester hydrolysis plus additional steps such as oxidative defluorination and hydroxylation. To conclude, the data showed that a hepatotoxicity of the investigated SC cannot be excluded, that metabolism seems to play a minor role in the observed effects, and that the extensive phase I metabolism is mediated by several isozymes making interaction unlikely.

**Supplementary Information:**

The online version contains supplementary material available at 10.1007/s00204-021-03148-3.

## Introduction

The ongoing emergence of new psychoactive substances (NPS) on the drugs of abuse market remains an analytical challenge for clinical and forensic toxicologists. Moreover, numerous case reports of intoxications or even deaths after intake of synthetic cannabinoids (SC) demonstrate the threat on public health (Adamowicz et al. 2019; Bolt and Hengstler 2021; Hvozdovich et al. 2020). Some reports associated their intake with liver failure, but knowledge about their hepatotoxic potential is sparse or even unknown (Solimini et al. 2017). Several in vitro strategies to assess the hepatotoxicity have been published using the hepatoma cell lines HepG2 or HepaRG, primary human hepatocytes, or rat hepatocytes (Dias da Silva et al. 2019; Luethi et al. 2017; O'Brien and Edvardsson 2017; Richter et al. 2019a; Roque Bravo et al. 2021). Most of them tested single cytotoxicity biomarkers in individual experiments, e.g., cell viability, collapse of mitochondrial membrane potential, increase of reactive oxygen or nitrogen species, damage on mitochondrial redox activity, leakage of lactate dehydrogenase (LDH) or cell death (Dias da Silva et al. 2019; Luethi et al. 2017; Roque Bravo et al. 2021). To prevent false-negative or -positive outcomes and to gain a deeper insight into intracellular processes, it is favorable to monitor multiple parameters at a subcellular level within the same experiment using high-content screening assays (HCSA) (O'Brien and Edvardsson 2017; Richter et al. 2019a). Advantages of HCSA include a high degree of automation, affordability, high sample throughput, analysis of multiple biomarkers during one run, as well as a higher sensitivity compared to the traditional assays (O'Brien and Edvardsson 2017; Richter et al. 2019a). However, the specificity of the HCSA is reduced compared to the “classical” assays (O'Brien and Edvardsson 2017; Richter et al. 2019a). In 2019, Richter et al. adopted a HCSA method to analyze the hepatotoxicity of nine different NPS including synthetic cathinones and the SC 5F-PB-22 in HepG2 cells. 5F-PB-22 was shown to possess the strongest cytotoxic characteristics of all investigated NPS (Richter et al. 2019a).

**Fig. 1:**
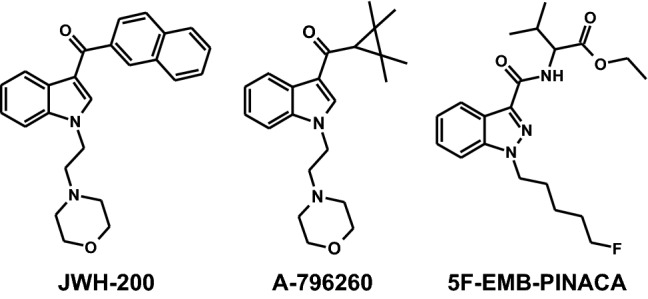
Structural composition of the investigated synthetic cannabinoids JWH-200, A-796260, and 5F-EMB-PINACA

However, as this HCSA method revealed some shortcomings regarding reproducibility, user friendliness, and sample throughput, the current study aimed to optimize the assay using a fully automated epifluorescence microscope. The optimized HCSA should then be used to study the cytotoxic properties of the SC JWH-200 (1-[2-(4-morpholinyl)ethyl]-3-(1-naphthoyl)-indole), A-796260 ([1-[2-(4-morpholinyl)ethyl]-indol-3-yl]-(2,2,3,3-tetramethylcyclopropyl)-methanone), and 5F-EMB-PINACA (ethyl-2-[[1-(5-fluoropentyl)-indazole-3-carbonyl]amino]-3-methyl-butanoate), which belong to different structural SC subclasses. JWH-200, an naphthoylindole class SC appeared on the market in 2009 (EMCDDA 2009), whereas the tetramethylcyclopropanoylindole compound A-796260 emerged in 2011 (Helander 2017). First reports about the indazole carboxamide type SC 5F-EMB-PINACA can be found in 2016 (DEA 2019). Structural compositions of investigated SC are given in Fig. 1. As not only parent compounds may cause hepatotoxic effects but also their metabolites (Dias da Silva et al. 2019; Roque Bravo et al. 2021), metabolism-based effects were also assessed. Moreover, the phase I metabolism of the SC was investigated, as knowledge about screening targets, crucial in analytical toxicology, is limited. The only exception is JWH-200, which has been investigated by De Brabanter et al. (2013) in pooled human liver microsomes (pHLM) and in urine of liver-humanized mice identifying 11 metabolites and isomers deduced therefrom. In the current study, also an isozyme mapping together with the identification of in vitro phase I metabolites was performed and analyzed by liquid chromatography–high-resolution tandem mass spectrometry (LC–HRMS/MS) to determine the involvement of single isozymes and initial metabolic steps to predict possible drug interactions.

## Materials and methods

### Chemicals and reagents

JWH-200, A-796260, and 5F-EMB-PINACA were purchased from Cayman Chemical (Ann Arbor, MI, USA) with a purity of 98.3% or higher. 5F-PB-22 was obtained from Lipomed (Weil am Rhein, Germany) and fluvastatin from LGC Standards (Wesel, Germany). 2- to 100-fold-concentrated stock solutions of JWH-200, A-796260, and 5F-EMB-PINACA were prepared in dimethyl sulfoxide (DMSO) and sterile filtered. Sterile stock solutions of Fluvastatin and 5F-PB-22 originated from a previous study (Richter et al. 2019a), which were also prepared 200-fold concentrated in DMSO. Hoechst33342, ionomycin, RPMI 1640 medium supplemented with GlutaMAX, sterile filters suitable for DMSO, and Tetramethylrhodamine, methyl ester (TMRM) were obtained from Life Invitrogen (Darmstadt, Germany). TOTO-3 was supplied by Thermo Fisher Scientific (Schwerte, Germany). HepG2 cells were obtained from the German collection of microorganism and cell cultures (DSMZ, Braunschweig, Germany). 1-Aminobenzotriazole (ABT), dipotassium hydrogen phosphate (K_2_HPO_4_), DMSO, ethylenediaminetetraacetic acid (EDTA), FCCP, isocitrate, isocitrate dehydrogenase, magnesium chloride (MgCl_2_), penicillin, poly-L-lysine (PLL), potassium dihydrogen phosphate (KH_2_PO_4_), superoxide dismutase, streptomycin, tris hydrochloride, Triton X-100, and trypsin were purchased from Sigma-Aldrich (Taufkirchen, Germany). Calcium chloride (CaCl_2_) and 4-(2-hydroxyethyl)-1-piperazineethanesulfonic acid (HEPES) were obtained by AppliChem (Darmstadt, Germany). Potassium chloride (KCl) was bought from Grüssing (Filsum, Germany). The 75 cm^2^ culture flasks were from Sarstedt (Nümbrecht, Germany). CAL-520 and NADP^+^ were obtained from Biomol (Hamburg, Germany). The 96-well half area high-content imaging plates, acetonitrile (LC–MS grade), methanol (LC–MS grade), ammonium formate (analytical grade), formic acid (LC–MS grade), and all other reagents and chemicals (analytical grade) were from VWR International (Darmstadt, Germany). The baculovirus-infected insect cell microsomes (Supersomes) containing human cDNA-expressed flavin-containing monooxygenase 3 (FMO3) (5 mg protein/mL), CYP1A2, CYP2A6, CYP2B6, CYP2C8, CYP2C19, CYP2D6, CYP3A4, CYP3A5 (1 nmol/mL), CYP2C9, and CYP2E1 (2 nmol/mL), as well as pHLM (20 mg microsomal protein/mL, 330 pmol total CYP/mg protein, 35 donors) and fetal bovine serum (FBS) were supplied by Corning (Amsterdam, The Netherlands). Enzymes were thawed at 37 °C, aliquoted, snap-frozen in liquid nitrogen, and stored at − 80 °C until use.

### Cell culture

As recently described by Richter et al. (2019a), HepG2 cultures were maintained at 37 °C with 95% humidity and 5% CO_2_ atmosphere in an incubator (Binder, Tuttlingen, Germany). Cells were handled under sterile conditions using a laminar flow bench class II (Thermo Fisher Scientific, Schwerte, Germany). According to the manufacturer’s recommendation, cells were cultivated in RPMI medium with 100 U/mL penicillin, 100 μg/mL streptomycin, and 10% (v/v) FBS (medium plus supplements) as additives within 75 cm^2^ culture flasks. PBS solution (137 mM NaCl, 2.7 mM KCl, 1.5 mM KH_2_PO_4_, 8.1 mM Na_2_HPO_4_, pH 7.4) and 0.05% (v/v) trypsin EDTA solution were used to passage cells every 3–4 days. Passage number 5 till 9 were used for all experiments. Single HepG2 stocks were cryopreserved and stored in liquid nitrogen at − 160 °C until use.

### Cell plate preparation

According to Richter et al. (2019a) and with minor modifications, 96-well half area high-content imaging plates were coated with PLL by adding 100 µL aqueous PLL solution (100 µg/mL) and incubation for 30 min under laminar air flow at room temperature (21 °C). Thereafter, plates were washed once with 150 µL autoclaved water and twice with 150 µL medium plus supplements. Cells were counted using a hemocytometer (Carl Roth, Karlsruhe, Germany). Afterwards, cells were seeded in a density of 1500 or 1750 cells/well by transferring 100 µL of the cell suspension to the precoated well plates. Cell plates were ready for drug treatment after incubation of 24 h at 37 °C with 95% humidity and 5% CO_2_ atmosphere.

### HCSA optimization and quality controls

As positive controls, the following reagents were used: mitochondrial uncoupler FCCP, calcium ionophore ionomycin, and membrane perturbing detergent Triton X-100. Fluvastatin and the SC 5F-PB-22 were used for method optimization and verification. All given concentrations in the following passages are final concentrations. Based on previous results, fluvastatin was incubated in four (0.14, 1.23, 3.7, and 11.1 µM) and 5F-PB-22 in seven concentrations (1.95, 3.91, 7.81, 15.6, 31.3, 62.5, and 125 µM) (Richter et al. 2019a). Before drug treatment, drug dilutions were freshly prepared in tubes using medium plus supplements. Then, 75 µL of the supernatants from the cell plates were removed and 50 µL of the drug solutions were added. Furthermore, each cell plate contained wells without drug but 0.5% (v/v) DMSO in medium plus supplements (blank incubations). Cell plates were incubated for either 48 h or 72 h at 37 °C with 95% humidity and 5% CO_2_ atmosphere. Afterwards, supernatants were removed, and cells were loaded with 75 µL of a fluorescence dyes cocktail consisting of 0.8 μM Hoechst33342, 1 μM CAL-520, 20 nM TMRM, and 1 μM TOTO-3 in medium plus supplements. After an incubation for another hour at 37 °C with 95% humidity, 5% CO_2_ atmosphere and protected from light, cells were washed three times with 50 µL ringer solution (140 mM NaCl, 2.8 mM KCl, 2 mM MgCl_2_, 1 mM CaCl_2_, 10 mM HEPES, pH 7.4), but the last 50 µL remained on the cells during measurement.

Negative controls were performed as described above by incubating cells in medium plus supplements (untreated), or additional 0.5% (v/v) DMSO. Each positive control was prepared in ringer solution in the following concentrations: FCCP, 100 µM; ionomycin, 10 µM; Triton X-100, 0.05% (v/v). After addition to the cell plate with the last washing step, positive controls remained on the cells during measurement. All incubations were done fivefold.

### Cytotoxicity (pre)screening and CYP inhibition

To assess the cytotoxicity of JWH-200, A-796260, and 5F-EMB-PINACA and potential metabolism-related effects, cell plates were prepared as described above, but incubations were conducted with final drug concentrations of 7.81 and 125 µM with or without 100 µM ABT. The given ABT concentration was based on the CYP inhibition in the HepaRG experiment by Yokoyama et al. (2018). Negative controls without test compound (blank incubations) but with additional ABT were performed to examine if one of the parameters were affected by ABT. If a strong or moderate cytotoxic potential was assigned, the SC was incubated again as defined below at seven concentrations (1.95, 3.91, 7.81, 15.6, 31.3, 62.5, and 125 µM) without ABT. All incubations were done fivefold.

### Microscope settings for HCSA

A Lionheart FX Automated Microscope (BioTek, BT, Bad Friedrichshall, Germany) equipped with an incubation chamber was used for cell plate analysis. During analysis, the incubation conditions were kept constant at 37 °C and lids were left on the cell plates to prevent evaporation. Images were recorded by a 20x/0.45 (semi-apochromat) objective. Hoechst33342 was captured in the first channel using the autofocus mode followed by all other dyes. Camera settings were as follows: LED value, 1 (Hoechst33342 and CAL-520) and 2 (TMRM and TOTO-3); camera gain, 24 dB; integration time (IT), Hoechst3342, 240 ms; TMRM, 1000–1550 ms; CAL-520, 200–564 ms; TOTO-3, 600–794 ms. Before each measurement, ITs were controlled using blank incubations and if necessary adapted. Each plate was analyzed within 60 min by collecting six images per well in two rows and three columns with a fixed distance in between. BT Gen5 Image Prime 3.09 software was used for data handling including image analysis.

### Image analysis

Prior to analysis, images were manually inspected for quality issues such as blurriness or artifacts by the nuclei fluorescence of Hoechst33342 and if necessary excluded. Number and area of regions identified by Hoechst33342, and its total fluorescence intensity were used to determine the cell count as measure for cell proliferation, nuclear size, and nuclear intensity. Mitochondrial membrane potential was determined by the total fluorescence intensity of TMRM, the fluorogenic calcium-sensitive dye CAL-520 intensity was used to identify cytosolic calcium levels. TOTO-3, which only binds to cellular DNA when the integrity of the plasma membrane is disrupted, was used to monitor plasma membrane integrity. For data normalization, an average value was calculated for each parameter per well using the total number of images after image inspection. Thereafter, each parameter except of the cell count was normalized to the cell count.

### Statistical analysis

Data of image analysis were exported to Microsoft Excel 2016 (Microsoft Corporation, WA, USA) to normalize the cell count to the number of appropriate images per well. A comparison between two different treatment groups was done using a two-tailed student’s test considering *P* values < 0.05 as statically significant (95% confidence interval). If more than two different treatment groups were compared to each other one-way ANOVA followed by either Dunnett’s or Tukey’s post hoc test was applied with a significance level of 0.05 (95% confidence interval). A Dunnett’s test was done to compare control data to all other experiment data, while a Tukey’s test compared all data with each other. Statistical analysis and *P* values were obtained by GraphPad Prism 5.00 (GraphPad Software, San Diego, CA, USA).

### Assessment of cytotoxic potential by prescreening

Assessment criteria for cytotoxicity were based on the study by Richter et al. (2019a) using adjusted concentrations as follows: a strong cytotoxic potential was defined when at least two parameters were significantly impaired at a low concentration (7.81 μM). A moderate cytotoxic potential was defined when two parameters were significantly affected and one of these at a concentration of 7.81 μM. Both ratings were considered as a positive result. A negative result was defined when parameters were only significantly influenced at a high concentration (125 μM).

### Isozyme mapping and in vitro phase I metabolism

Incubation conditions followed an established protocol (Wagmann et al. 2016). Details are outlined in the Electronic Supplementary Material (ESM).

### LC–HRMS/MS apparatus for identification of metabolites

Analysis was performed according to a previously published study (Gampfer et al. 2019) and further details can be found in the ESM.

## Results

### Method optimization

**Fig. 2:**
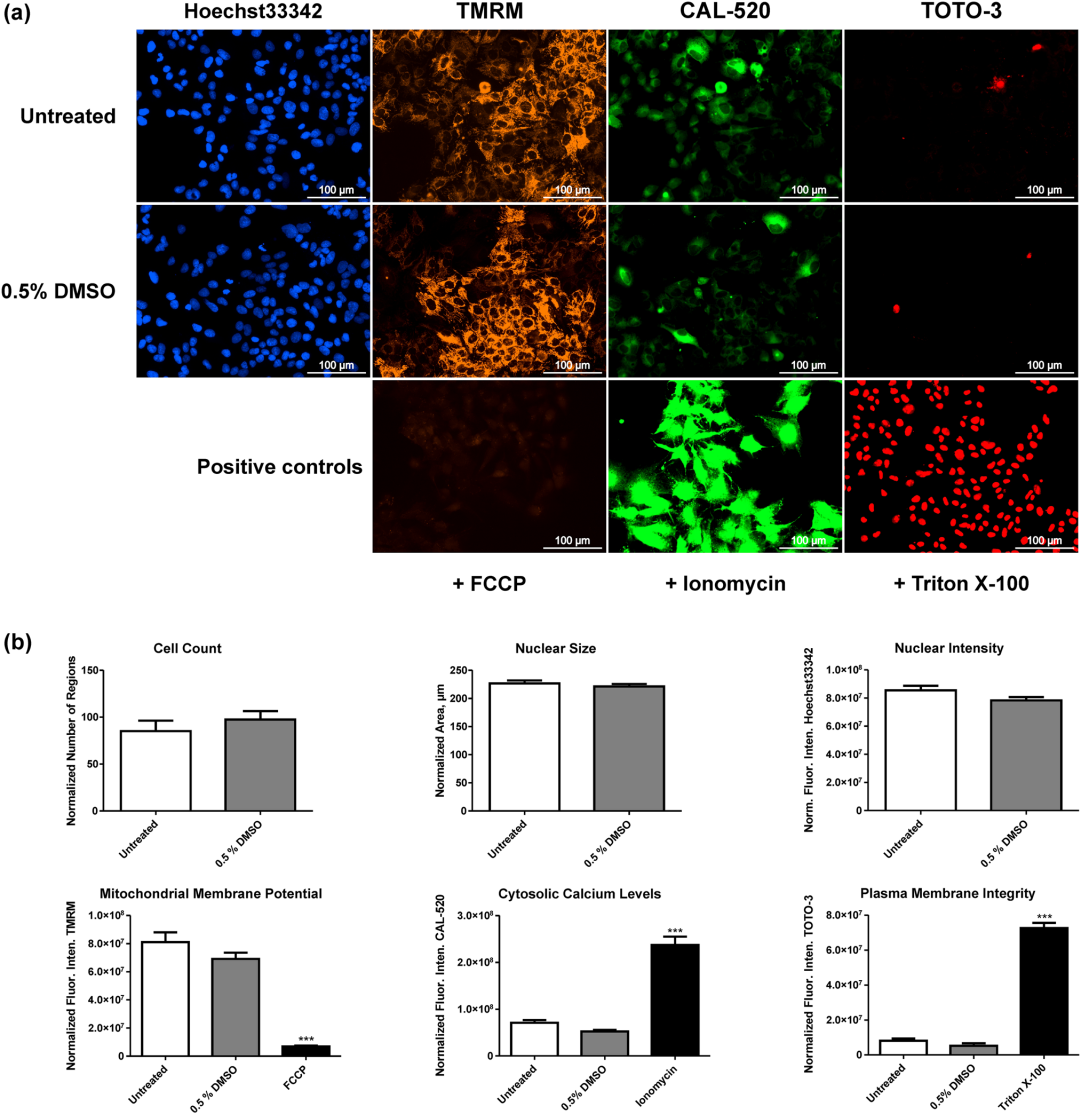
Fluorescence microscopic images of cells incubated with Hoechst33342, TMRM, CAL-520, and TOTO-3 (**a**) and measured effects on the six parameters (cell count, nuclear size, nuclear intensity, mitochondrial membrane potential, cytosolic calcium levels, and plasma membrane integrity) (**b**) obtained by incubation of cells without treatment, with 0.5% DMSO, and the corresponding positive control (FCCP, ionomycin, or Triton X-100). All parameters were normalized to the cell count except for the cell count, which was normalized to the number of appropriate images. Values are expressed as mean ± standard error of the mean (SEM; *n* = 5). Statistical analysis was done using either a *t*-test or one-way ANOVA followed by Dunnett’s post hoc test (***, *P* < 0.001 compared to untreated cells). Norm. Fluor. Inten., normalized fluorescence intensity

Quality control incubations were performed to exclude any effects derived from 0.5% DMSO on the tested parameters and to check the maximal or minimal fluorescence response of each dye. As positive controls, FCCP a protonophore and mitochondrial uncoupler, ionomycin a calcium ionophore, and Triton X-100 a membrane perturbing detergent were used. Figure 2a shows images of HepG2 cells stained with Hoechst33342, TMRM, CAL-520, and TOTO-3 and Fig. 2b measured effects on the six different parameters after incubation of untreated cells, treated with 0.5% DMSO or with a corresponding positive control. Comparisons between untreated cells and cells treated with 0.5% DMSO showed no statistically significant differences in all six investigated parameters, whereas cells treated with one of the positive controls showed significant differences compared to untreated cells. As expected, a decreasing fluorescence intensity of TMRM was observed in incubations with FCCP and an increasing fluorescence intensity of CAL-520 and TOTO-3 in incubations with ionomycin and Triton X-100, respectively.

Dose–response plots of the six parameters after incubation with fluvastatin (a) and 5F-PB-22 (b) are shown in Fig. S1 in the ESM. As expected, fluvastatin significantly decreased the cell count from a concentration of 1.23 µM in comparison to blank incubations. Although no significance could be stated for the other five parameters, a general trend is apparent except for the plasma membrane integrity. Thus, changes manifest in a decrease of the nuclear size, a hyperpolarization of the mitochondrial membrane, and an increase of the nuclear intensity by chromatin condensation and a rise of intracellular calcium levels.

Referring to 5F-PB-22, significant variations of treated cells were observed by a decrease of the cell count from a concentration of 15.63 µM, the nuclear intensity from 1.95 µM, the mitochondrial membrane potential from 1.95 µM and the cytosolic calcium from 31.25 µM compared to blank incubations, respectively. However, the nuclear size remained unaffected by 5F-PB-22 and although the overall trend of the plasma membrane integrity decreased through increasing TOTO-3 fluorescence the different values were highly variable.

### Cytotoxicity (pre)screening with metabolism-based effects

**Fig. 3:**
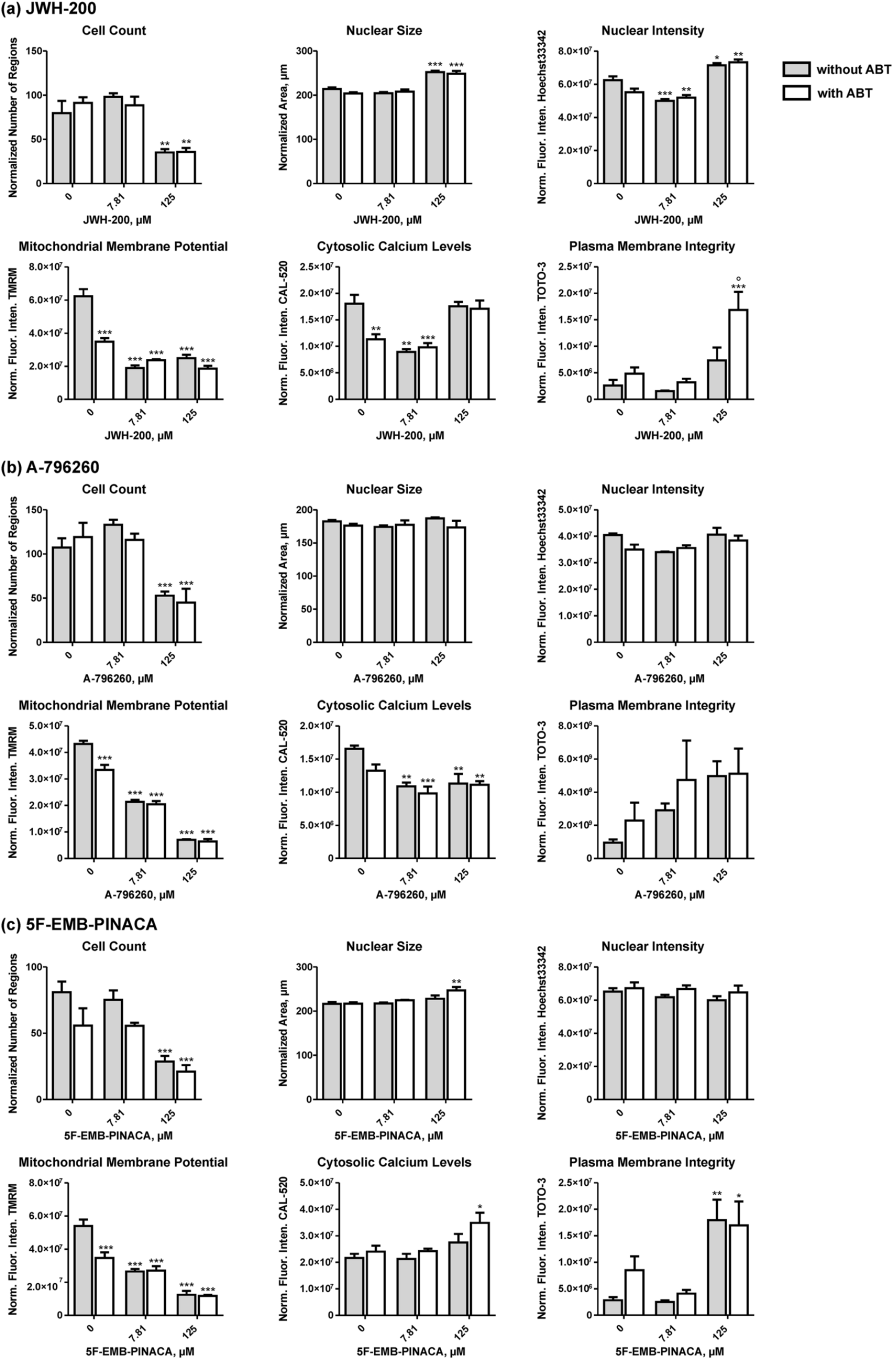
Dose–response plots of JWH-200 (**a**), A-796260 (**b**), and 5F-EMB-PINACA (**c**) obtained by incubation at two different concentrations (7.81 and 125 µM) and blank incubations without test compound with and without the cytochrome P450-inhibitor 1-aminobenzotriazole (ABT), respectively. Changes of different parameters (cell count, nuclear size, nuclear intensity, mitochondrial membrane potential, cytosolic calcium levels, and plasma membrane integrity) are plotted in relation to blank incubations (100%). All parameters were normalized to the cell count except the cell count, which was normalized to the number of appropriate images. Values are expressed as mean ± standard error of the mean (SEM; n = 5). Statistical analysis was done using one-way ANOVA followed by Tukey’s post hoc test (***P < 0.001, **P < 0.01, *P < 0.05 compared to blank incubation; °*P* < 0.05 compared to incubations with identical treatment without ABT). Norm. Fluor. Inten., normalized fluorescence intensity

Results of the cytotoxicity prescreening are presented in Fig. 3a–c, which were determined by incubation of each JWH-200, A-796260, and 5F-EMB-PINACA at concentrations of 7.81 µM and 125 µM with or without the CYP inhibitor ABT. Based on the criteria defined above, a strong cytotoxic potential was assigned for JWH-200 and A-796260, whereas 5F-EMB-PINACA showed only a moderate potential. Incubations with additional ABT showed no significant effects compared to incubations at equal SC concentrations but without ABT except for JWH-200 at 125 µM, which adversely affected the plasma membrane integrity. For negative control incubations containing ABT but no SC, a reduced mitochondrial membrane potential and in case of the negative control of JWH-200 also decreased intracellular calcium levels were observed compared to blank incubations.

**Fig. 4:**
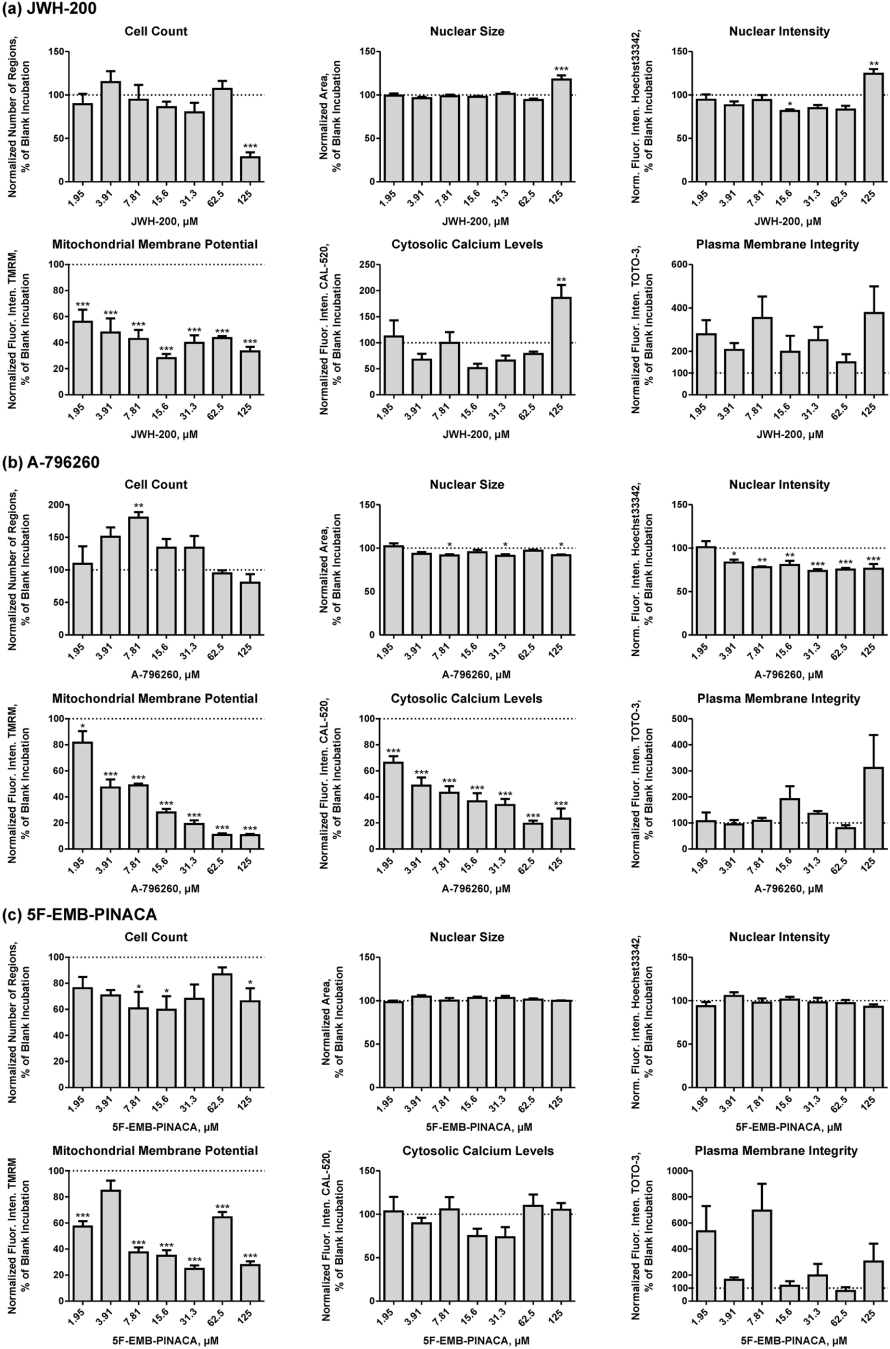
Dose–response plots of JWH-200 (**a**), A-796260 (**b**), and 5F-EMB-PINACA (**c**) obtained by incubation of cells at seven concentrations of the respective test compound (1.95, 3.91, 7.81, 15.6, 31.3, 62.5, and 125 µM) and blank incubations without test compound. Changes of cell parameters (cell count, nuclear size, nuclear intensity, mitochondrial membrane potential, cytosolic calcium levels, and plasma membrane integrity) are plotted in relation to blank incubations (100%). All parameters were normalized to the cell count except the cell count, which was normalized to the number of appropriate images. Values are expressed as mean ± standard error of the mean (SEM; *n* = 5). Statistical analysis was done using one-way ANOVA followed by Dunnett’s post hoc test (***P < 0.001, **P < 0.01, *P < 0.05 compared to blank incubation). Norm. Fluor. Inten., normalized fluorescence intensity

Results of the cytotoxicity screening after incubation of JWH-200 (a), A-796260 (b), and 5F-EMB-PINACA (c) in seven concentrations from 1.95 to 125 µM are given in Fig. 4. JWH-200 significantly reduced the cell count and mitochondrial membrane potential at a concentration of 125 and 1.95 µM, respectively. Furthermore, the nuclear size, nuclear intensity, and intracellular calcium levels increased at 125 µM. Whereas the plasma membrane integrity showed a high variability with no significant changes. A-796260 significantly impaired the nuclear intensity at 3.91 µM and the mitochondrial membrane potential and cytosolic calcium levels at 1.95 µM, respectively. Initially, the cell count increased at 7.81 µM; however, afterwards a downward trend was observed. Besides, A-796260 barely affected the nuclear size and the increase of TOTO-3 fluorescence intensity was not significant. In case of 5F-EMB-PINACA, at first the cell count was significantly reduced at 7.81 µM, but then reversed with increasing concentration and declined again at 125 µM. Similar variations were also detected for the mitochondrial membrane potential, which first decreased at 1.95 µM followed by an increase and renewed reduction at 7.81 µM. No significance could be determined for the nuclear size, nuclear intensity, intracellular calcium levels, and plasma membrane integrity after treatment with 5F-EMB-PINACA.

### In vitro phase I metabolites

All detected phase I metabolites of JWH-200, A-796260, and 5F-EMB-PINACA in pHLM or isozyme incubations along with their metabolite identification number, the calculated exact mass of the protonated molecule, elemental composition, retention time, and the three most abundant fragment ions (FIs) recorded in the HRMS^2^ mode are given in Table S1–S3 in the ESM, respectively.

All incubations including negative controls of each JWH-200 and A-796260 contained one hydroxy-metabolite (MA21 and MB17) with similar signal intensities, thus both were most likely of an artificial origin. In negative control incubations of 5F-EMB-PINACA, no degradation products were detected. In the current study, 28 phase I metabolites of JWH-200 were found in all incubations. Observed metabolic reactions included a *N*-dealkylation (MA1), together with hydroxylation (MA4), oxidative morpholine cleavage (MA2), and additional oxidation to carboxylic acid (MA3), or hydroxylation (MA5–MA7), or dihydrodiol formation by epoxidation and non-enzymatic hydrolysis (MA8). Further metabolites were generated by oxidative opening of the morpholine ring (MA9, MA12, and MA13), together with either hydroxylation (MA10, MA11, and MA24), or dihydrodiol formation (MA14). Other biotransformation steps resulted in a hydroxylation and subsequent oxidation to a ketone (MA15 and MA17), dihydroxylation followed by dehydrogenation (MA16), hydroxylation (MA18–MA23) followed by dehydrogenation and dihydrodiol formation (MA25), dihydroxylation (MA25) as well as dihydrodiol formation (MA27 and MA28). The four most abundant metabolites in pHLM incubations were *N*-dealkylated (MA1), oxidative morpholine cleaved (MA2), and oxidative morpholine opened (MA9 and MA13).

In case of A-796260, a total number of 22 phase I metabolites were identified, which originated partly from a *N*-dealkylation (MB1) and additional hydroxylation (MB3), oxidative morpholine cleavage (MB2), also together with hydroxylation (MB4 and MB5), oxidative morpholine opening (MB6, MB9, and MB10) plus hydroxylation (MB7, MB19, and MB20). Moreover, metabolites were formed by hydroxylation followed by dehydrogenation (MB8), plus additional hydroxylation (MB12), or oxidation (MB13), dihydroxylation and subsequent dehydrogenation (MB11), hydroxylation (MB14–MB18), and dihydroxylation (MB21 and MB22). The four most abundant metabolites in pHLM incubations were as follows: oxidative morpholine cleavage (MB2), oxidative morpholine opening (MB6), dihydroxylation followed by dehydrogenation (MB11), and hydroxylation (MB14).

Concerning 5F-EMB-PINACA, 23 metabolites were detected, which originated from a *N*-dealkylation (MC2) and simultaneous ester hydrolysis (MC1) or together with hydroxylation (MC3 and MC4), ester hydrolysis and oxidative defluorination (MC5) followed by further oxidation to carboxylic acid (MC8), and lactone formation with or without previous ester hydrolysis (MC6). Other specified metabolites included the ester hydrolysis (MC7) along with hydroxylation (MC9–MC11), oxidative defluorination (MC12) either together with hydroxylation and oxidation to a ketone (MC14) or subsequent oxidation to carboxylic acid (MC15). Besides, metabolites were generated by hydroxylation (MC16–MC18) followed by dehydrogenation (MC13) or oxidation to carboxylic acid (MC20), as well as dihydroxylation (MC21–MC23) followed by oxidation to a ketone (MC19). The four most abundant metabolites in pHLM incubations included metabolites generated by ester hydrolysis (MC7) plus additional metabolic steps such as oxidative defluorination (MC5), oxidative defluorination to carboxylic acid (MC8), and hydroxylation (MC9).

### Isozyme mapping of initial phase I steps

Results of the isozyme mapping of initial phase I metabolites in comparison to pHLM incubations of JWH-200, A-796260, and 5F-EMB-PINACA are summarized in Table S4–S6 in the ESM.

*N*-Dealkylation (MA1) of JWH-200 was catalyzed by CYP1A2, CYP3A4, and CYP3A5, whereas the metabolite generated by oxidative morpholine cleavage (MA2) was only found in CYP3A4 and CYP3A5 incubations. Several isozymes were involved on the oxidative morpholine ring opening (MA9, MA12, and MA13), namely CYP1A2, CYP2B6, CYP2C8, CYP2C19, CYP2D6, CYP3A4, and CYP3A5. All aforementioned isozymes also contributed to the formation of the monohydroxy metabolites (MA18–MA23) but CYP2B6 was replaced by CYP2C9. Metabolites formed by a dihydrodiol formation (MA27 and MA28) were detected in CYP1A2, CYP2C19, CYP3A4, and CYP3A5 incubations.

Similar to JWH-200, *N*-dealkyl-A-796260 (MB1) and the metabolite formed by oxidative morpholine cleavage (MB2) were both formed by CYP3A4 and CYP3A5 and additional by CYP1A2 in case of *N*-dealkyl-A-796260 (MB1). Furthermore, the isozymes which contributed to the oxidative morpholine ring opening of A-796260 (MB6, MB9, and MB10) were in accordance with that of JWH-200 except for CYP2B6. Monohydroxy metabolites (MB14–MB18) were formed by various isozymes including CYP1A2, CYP2C8, CYP2C9, CYP2D6, CYP3A4, and CYP3A5.

The following isozymes were involved on the formation of *N*-dealkylated 5F-EMB-PINACA (MC2): CYP1A2, CYP2B6, CYP2C19, CYP3A4, and CYP3A5. The ester hydrolysis product (MC7) was the only initial metabolite detected in positive control incubations. Moreover, metabolic reactions such as the oxidative defluorination (MC12) or hydroxylation (MC16–MC18) were in turn catalyzed by numerous isozymes. The former metabolite (MC12) was present in incubations of CYP1A2, CYP2B6, CYP2C8, CYP2C9, CYP2C19, CYP2D6, and CYP3A5. These isozymes also contributed to the hydroxylation of 5F-EMB-PINACA (MC16–MC18) except for CYP2D6 which was substituted by CYP3A4.

## Discussion

### HCSA optimization

To overcome some drawbacks of a previously developed HCSA method (Richter et al. 2019a) such as reproducibility, user friendliness, and sample throughput, an optimized HCSA method should first be developed. With the improved HCSA method using a fully automated epifluorescence microscope, all parameters could be analyzed for statistical significance. Although O'Brien and Edvardsson (2017) mentioned, that at least 3 days of cell treatment with test compounds is needed for HepG2 cytotoxicity testing, it was necessary to reduce the treatment from 72 to 48 h to prevent cells from unpredictable cell growth such as multilayer formations. Furthermore, work load and duration of the experiment could thus be reduced. To prove the suitability of the method, two formerly tested compounds showing a high cytotoxic potential, fluvastatin and 5F-PB-22, were reanalyzed (Richter et al. 2019a). Statin induced hepatotoxicity is still not fully understood, but various triggers have been debated such as blockage of the cholesterol biosynthesis, decreasing levels of isoprenoids, e.g., ubiquinone and quite likely the inhibition of the 3-Hydroxy-3-methyglutaryl-coenzyme A reductase leading to reduced formation of mevalonate and its metabolites such as farnesol and geranylgeraniol (Diaz and O’Brien 2006). In the previous study, fluvastatin significantly affected the cell count and nuclear size at concentration of 11.12 µM and the nuclear intensity at 100 µM. In general, the remaining three parameters, namely mitochondrial membrane potential, cytosolic calcium levels, and plasma membrane integrity were not tested for significance. However, the mitochondrial membrane potential was reduced with increasing concentration, cytosolic calcium levels showed a peak at 11.12 µM and the plasma membrane integrity was inconsistent (Richter et al. 2019a). Concerning the present study, only the cell count showed a statistical significance, which could be due to the high variances induced by the strongly reduced cell count since all other parameters were normalized to cell count. As a consequence, the seeded cell number was adjusted from 1500 to 1750 cell per well for all subsequent tests. The prior 5F-PB-22 experiment revealed a significantly decreased cell count and nuclear intensity at 1.95 µM and nuclear size at 3.9 µM. Mitochondrial membrane potential was reduced with increasing 5F-PB-22 concentration, whereas cytosolic calcium levels and plasma membrane integrity were increased (Richter et al. 2019a). Similar to Richter et al. (2019a) the cell count and mitochondrial membrane potential decreased in the current assay. In case of the nuclear intensity, initially the fluorescence intensity was reduced as determined by Richter et al. (2019a); however, it increased at a concentration of 125 µM. The decreased nuclear intensity at lower concentrations could be explained by an intercalation of 5F-PB-22 into DNA, as already reported for other drugs such as doxorubicin (O'Brien and Edvardsson 2017). As Hoechst33342 is a DNA-binding fluorescence dye, Hoechst33342 and 5F-PB-22 compete for the binding site at the DNA. The elevated nuclear intensity at a high concentration could be reasoned by chromatin condensation caused by DNA degradation (O'Brien and Edvardsson 2017). In the present study, the nuclear size after treatment of 5F-PB-22 was barely impaired and the plasma membrane integrity declined rather unstable through increased fluorescence intensity. Those divergent results in comparison to the previous study (Richter et al. 2019a) might be explained by the reduced treatment time. Contrary to Richter et al. (2019a) the cytosolic calcium levels of 5F-PB-22 decreased, which has not yet been described for SC in literature, except for neuronal cells (Zhuang et al. 2005). Another explanation could be the presence of active transporters in living cells removing CAL-520 from the cytosol as postulated by Richter et al. (2019a).

### Cytotoxicity of the tested synthetic cannabinoids

Two different evaluation strategies to assess the cytotoxic potential of drugs and/or NPS following a HCSA were described in the literature (O'Brien and Edvardsson 2017; Richter et al. 2019a). The approach developed by O´Brien and Edvardsson (2017) depends on known maximum blood concentrations (*C*_max_) values, which are usually unavailable, due to the lack of controlled studies for ethical reasons. Moreover, no plasma concentrations or consumer dosages have yet been described. Solely for JWH-018, a JWH-200 derivative, *C*_max_ values after inhalation of a 2 or 3 mg dose were published ranging from 0.009 to 0.03 µM (Toennes et al. 2017). Therefore, a first cytotoxicity estimation was done using a prescreening at a low and high concentration (7.81 and 125 µM) following the procedure proposed by Richter et al. (2019a). Since the result of the pretest was positive for all three SC, they were again incubated at seven different concentrations (1.95–125 µM) to gain a deeper insight into cytotoxic effects. To date, none of the investigated SC was analyzed for its hepatotoxicity. With regard to hepatotoxic properties of structural related SC, only a few studies are available (Giuliano et al. 2009; Koller et al. 2013; Richter et al. 2019a). Apart from 5F-PB-22, which showed a strong cytotoxic potential (Richter et al. 2019a), the morpholino derivate WIN 55,212-2 was examined in HepG2 cells (Giuliano et al. 2009). In this study cell viability using a 3-(4,5-dimethylthiazol-2-yl)-2,5-diphenyltetrazolium bromide reduction assay, mitochondrial membrane potential using 3,3-dihexyloxacarbocyanine, and morphological apoptotic changes using Hoechst33258 were investigated among others (Giuliano et al. 2009). WIN 55,212-2 concentrations ranged from 1 to 10 µM and cells were treated for 16, 24, 36, 48, or 72 h depending on the assay. Effects on the cell viability (–25%) were already observed after 24 h of treatment with 10 µM WIN 55,212-2, which increased with treatment time and was about 10% of control cells after 72 h (Giuliano et al. 2009). Collapse of mitochondrial membrane potential was induced after 16 h of treatment with 10 µM and increased with treatment time (Giuliano et al. 2009). Furthermore, at 36 h of treatment with 10 µM WIN 55,212-2, an increased number of cells were observed showing typical apoptotic features such as condensed or fragmented nuclei (Giuliano et al. 2009). In another work by Koller et al. (2013), naphthylindole compounds such as JWH-018, JWH-073, JWH-122, and JWH-210 were investigated in HepG2 cells among others using a LDH leakage assay and changes of mitochondrial functions were assessed by alterations of the succinate dehydrogenase activity of the cells with the 2,3-bis(2-methoxy-4-nitro-5-sulfophenyl)-5-[(phenyl-amino)carbonyl]-2H-tetrazolium hydroxide (XTT) assay (Koller et al. 2013). No damage on the cell membrane of HepG2 cells by LDH leakage could be determined after a 24 h treatment for all investigated SC (10–100 µM). Whereas, JWH-018 and JWH-122 were positive in the XTT assay in HepG2 cells (Koller et al. 2013). Thus, data about a potential hepatotoxicity of SC are still limited and the current study partly closes this gap by providing insights into the cytotoxic effect of some SC belonging to different subclasses. JWH-200 and A-796260 show some structural similarities such as the 4-morpholinylethyl moiety linked to the indole group. However, JWH-200 consisted of a naphthyl head group, which is substituted by a 2,2,3,3-tetramethylcyclopropyl (TMCP) in case of A-796260. By contrast, 5F-EMB-PINACA, which is composed of a fluoro pentyl chain and an indazole core linked to an ethyl-methylbutanoate moiety, showed no structural overlap with the aforementioned SC. According to the criteria given above, a strong cytotoxic potential was assigned for the naphthylindole JWH-200 and the tetramethylcyclopropanoylindole A-796260, whereas the indazole carboxamide 5F-EMB-PINACA showed only moderate cytotoxic effects. Overall, the results of the pretest were in close consistency with those of the subsequent screening except for few parameters, which showed high variances or an unexpected outcome. Contradictory results of the cell count may be due to inaccuracies during cell seeding. In part, the calcium levels showed substantial differences and similarly to 5F-PB-22, A-796260 revealed an unexpected reduction of CAL-520 fluorescence intensity, which were already discussed above. However, there was no plausible explanation for variations of the plasma membrane integrity, hence this parameter seems less suited as a reliable indicator of cytotoxicity. In addition, the nuclear size appears rather inadequate as the automated software had sometimes difficulties in distinguishing two or more nuclei from each other. Nonetheless, a general recommendation to not consider parameters such as the plasma membrane integrity in the future might not be given, as this should be first evaluated on further NPS classes. As already reported by Richter et al. (2019a), the mitochondrial potential was found to be the most sensitive parameter, and therefore, the best choice to monitor a potential cytotoxicity.

Some reports about possible SC induced liver injuries can be found in the literature (Abouchedid et al. 2016; Shahbaz et al. 2018; Sheikh et al. 2014). However, cannabinoid receptor type 2 (CB2) agonists, as it is the case for A-796260 (Yao et al. 2008), have been associated with liver diseases such as obesity-associated inflammation and insulin resistance (Sherpa et al. 2015). In general, an elevated risk of liver injuries is assumed for consumers with intake of high doses or with additional risk factors. Considering that the latest generations of SC show a higher potency and efficiency at the in vitro CB_1_ receptor or CB_2_ as older ones (Krotulski et al. 2021; Lie et al. 2021), such as JWH-200 or A-796260, lower consumer doses of more recent compounds might be expected. One might also suppose, that SC concentrations in blood do not generally reflect their concentrations in hepatocytes, based on different blood and liver contents of the JWH-200 homolog JWH-210 in a pig model (Schaefer et al. 2017).

### Metabolism-based effects on the cytotoxicity

The hepatotoxicity of NPS may be mediated not only by the parent compounds but also by their metabolites, thus the metabolism-related impact was investigated using the broad CYP inhibitor ABT (Dias da Silva et al. 2019; Roque Bravo et al. 2021). Based on the current findings of the isozyme mapping, various isozymes were involved in the SC metabolism and the use of single isozyme inhibitors appeared not to be appropriate. Since only the plasma membrane integrity seemed impaired after treatment with ABT and JWH-200 in a high concentration, the outcome might indicate that the formed metabolites might be less toxic than the corresponding parent compounds. Another reason might be that HepG2 cells are known for their limited metabolic ability due to low gene expression of CYP enzymes (Hart et al. 2010). However, a few studies have been reported, that metabolites are detectable in HepG2 cell incubates (Richter et al. 2019a, 2017; Wagmann et al. 2021). To substantiate these assumptions, HepG2 incubates should have been analyzed for SC metabolites, which represents the main limitation of the current study.

However, according to a comparative study for drug metabolism-based hepatotoxic effects in HepG2, HepaRG, and primary human hepatocytes by Yokoyama et al. (2018), HepaRG cells should be the most suitable ones for analysis of metabolism-based cytotoxicity. Unlike primary human hepatocytes, HepaRG showed a stable expression of CYP enzymes but this major drawback of inter-individual variability of primary human hepatocytes could be overcome using pooled human hepatocytes (Yokoyama et al. 2018). Furthermore, a recent study revealed considerable differences between HepaRG and primary human hepatocytes among others with respect to CYP enzymes and transporter levels (Hammer et al. 2021). Alternatively, primary rat hepatocytes have been used to examine metabolism mediated effects (Dias da Silva et al. 2019; Roque Bravo et al. 2021) but the predictions of human toxicity deduced from mammalian cells have been reported to be lower than human cell lines in HCSA (O'Brien and Edvardsson 2017).

### Identification of metabolites

**Fig. 5:**
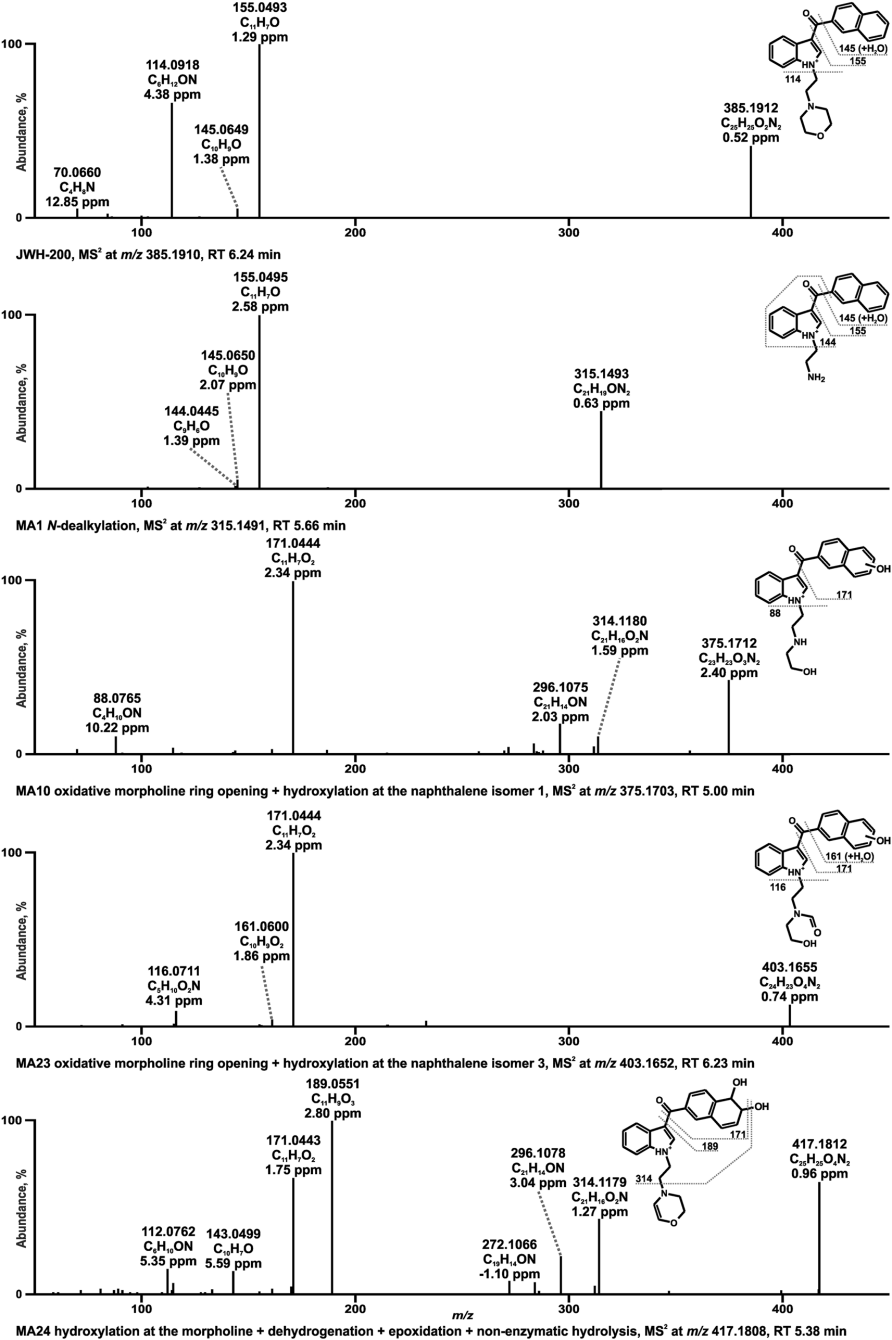
HRMS^2^ spectra of JWH-200 and selected metabolites identified in pooled human liver microsomes or isozyme incubations. Metabolites are ordered by increasing mass and retention time (RT). Metabolite IDs correspond to Table S1. JWH-200 metabolite (MA)

**Fig. 6:**
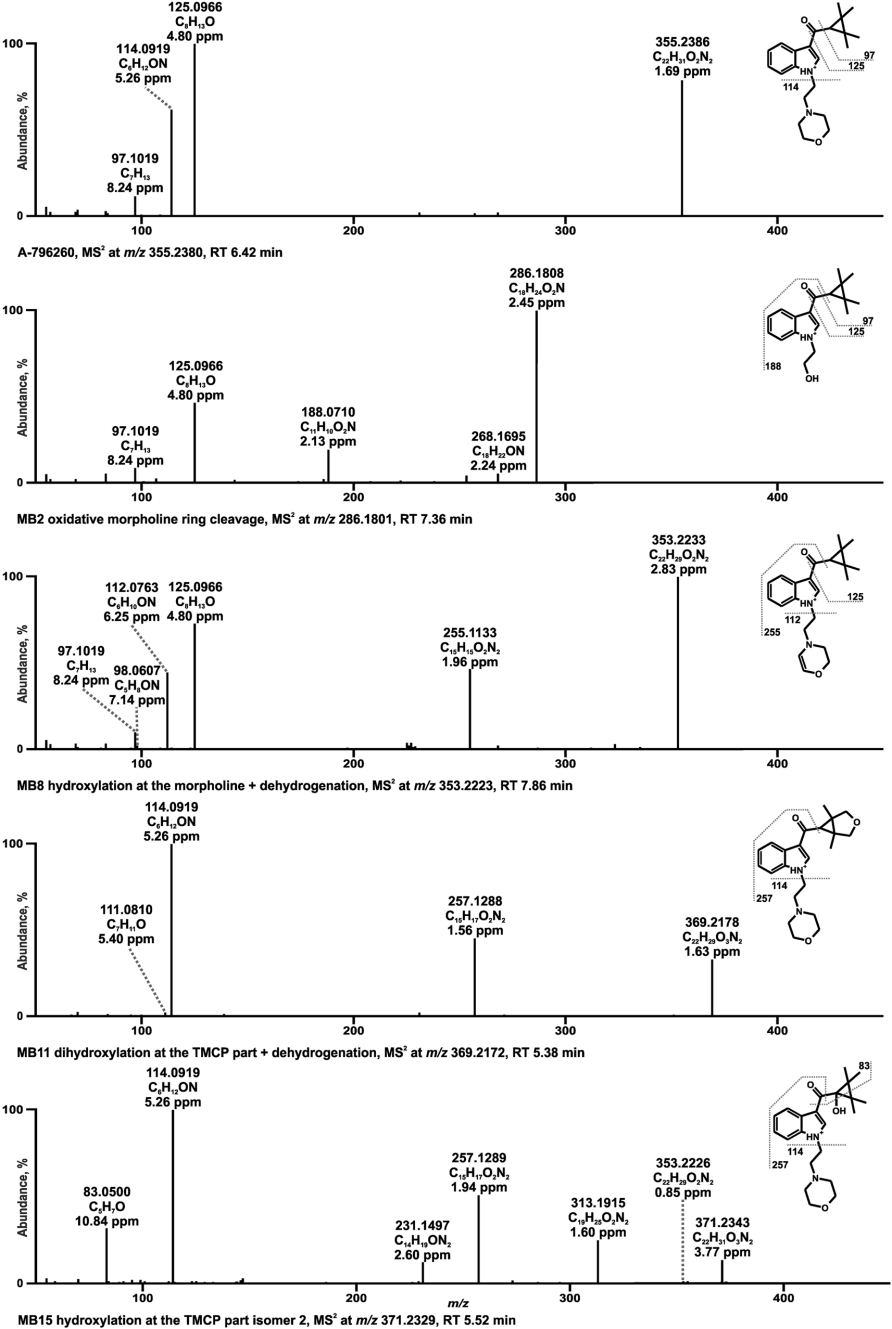
HRMS^2^ spectra of A-796260 and selected metabolites identified in pooled human liver microsomes or isozyme incubations. Metabolites are ordered by increasing mass and retention time (RT). Metabolite IDs correspond to Table S2. A-796260 metabolite (MB), tetramethylcyclopropyl (TMCP)

**Fig. 7:**
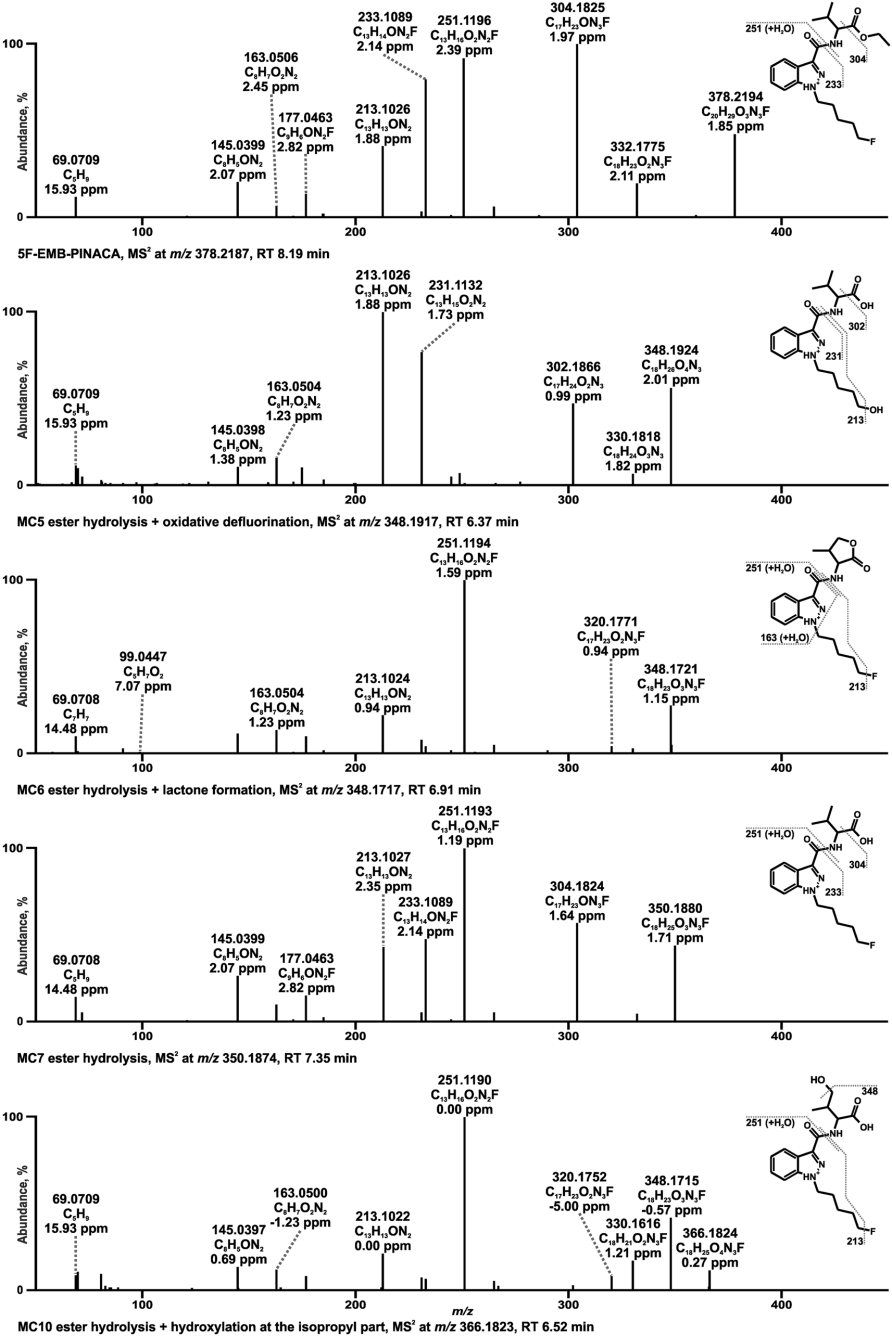
HRMS^2^ spectra of 5F-EMB-PINACA and selected metabolites identified in pooled human liver microsomes or isozyme incubations. Metabolites are ordered by increasing mass and retention time (RT). Metabolite IDs correspond to Table S3. 5F-EMB-PINACA metabolite (MC)

Even though investigations of metabolism-related effects in the HCSA indicated the formation of less toxic metabolites in HepG2 cells for all SC, identification of metabolites is crucial to figure out potential screening targets essential in analytical toxicology. Metabolites were tentatively identified by comparison of HRMS^2^ spectra and fragmentation patterns of the parent compounds to those of putative metabolites. Owing to the large number of detected metabolites, some representatives were selected to exemplify the general identification approach using the exact, calculated masses. HRMS^2^ spectra of JWH-200, A-796260, and 5F-EMB-PINACA and their metabolites, which are discussed in detail in the following, are given in Figs. 5, 6 and 7, respectively. All other HRMS^2^ spectra of identified metabolites are depicted in Fig. S2 (JWH-200), S3 (A-796260), and S4 (5F-EMB-PINACA) in the ESM.

### JWH-200

In case of JWH-200, a general comparison between metabolites identified in the current study and by De Brabanter et al. is hardly possible due to variations in pHLM reaction conditions such as an extended incubation time and elevated substrate and enzyme concentrations, sparse availability of HRMS^2^ spectra, and different sample preparation steps such as additional solid phase extraction (De Brabanter et al. 2013). However, differences turned out in the non-detectability of metabolites formed by hydroxylation and subsequent dehydrogenation at the morpholine and by hydroxylation at the morpholine plus dihydrodiol formation after epoxidation and non-enzymatic hydrolysis at the naphthalene. As a note, none of them was found in the urine of the liver-humanized mice (De Brabanter et al. 2013). Additional steps were identified by previously undescribed precursor masses such as the *N*-dealkylation (MA1), together with hydroxylation at the naphthalene (MA4), oxidative morpholine ring opening plus hydroxylation also at the naphthalene (M10 and M11), oxidative morpholine cleavage plus dihydrodiol formation (MA14), hydroxylation at the morpholine followed by dehydrogenation together with dihydrodiol formation (MA24), and dihydroxylation at the morpholine or ethyl part (MA26).

The fragmentation pattern of JWH-200 with a protonated precursor ion (PI) at *m/z* 385.1910 showed two high abundant FIs at *m/z* 155.0491 and at *m/z* 114.0913. The former one corresponded to the naphthalene with vicinal carbonyl group after elimination of the indole moiety and the latter FI belonged to the morpholine plus ethyl spacer formed by cleavage of the indole at the indole’s nitrogen atom. A lower abundant FI at *m/z* 145.0647 derived from FI at *m/z* 155.0491 by a loss of CO and addition of water. In addition, the FI at *m/z* 70.0651 resulted from FI *m/z* 114.0913 by opening of the morpholine ring and elimination of the C_2_ H_4_O group (− 44.0262 u).

The HRMS^2^ spectrum of *N*-dealkyl-JWH-200 (MA1) with PI at *m/z* 315.1491 contained two FIs with *m/z* at 155.0491 and at 145.0647, which were both consistent with those of the parent compound. However, their spectra differed in the low abundant FI at *m/z* 144.0647, which was only present in the spectrum of MA1. This fragment was composed of the indole ring plus carbonyl moiety by cleavage of the naphthalene. Two of three isomers formed by oxidative ring opening and hydroxylation (M10 and M11) with PI at *m/z* 375.1703 indicated a loss of an ethylene at the morpholine, which was confirmed by FI at *m/z* 88.0756 comprising the remaining part of the morpholine linked to the ethyl group. Whereas the third one (MA23) with PI at *m/z* 403.1652 was identified by FI at *m/z* 116.0706, which correlated with FI at *m/z* 114.0913 by loss of a methylene plus one additional oxygen atom. Although the oxidative ring opening mechanism at the morpholine ring is not known in detail it could be explained by the speculative pathway described by Denissen et al. (1994). Based on the FI at *m/z* 171.0440 in the spectra of all three isomers, which corresponded to FI at *m/z* 155.0491 by a shift of one oxygen (+ 15.9949 u) the hydroxylation occurred at the naphthalene. One metabolite generated by hydroxylation followed by dehydrogenation and dihydrodiol formation with PI at *m/z* 417.1808 (MA24) was characterized first by FI at *m/z* 189.0551 representing a dihydrodiol formation at the naphthalene plus vicinal carbonyl. This FI also derived from the aforementioned FI at *m/z* 155.0491 altered by two more oxygen and hydrogen atoms. Second, FI at *m/z* 112.0756 suggested a loss of water after a hydroxylation at the morpholine by a shift of two hydrogen atoms compared to FI at *m/z* 114.0913.

### A-796260

In the spectrum of A-796260 with the PI at *m/z* 355.2380 a prominent FI at *m/z* 125.0960 was detected, originating from the TMCP ring attached to the vicinal carbonyl group after elimination of the indole part. In accordance with JWH-200, FI at *m/z* 114.0913 was generated by the separation of the indole ring linked to the TMCP consisting of the morpholine plus ethyl spacer. Furthermore, the loss of CO (− 27.9949 u) from FI at *m/z* 125.0960 led to the FI at *m/z* 97.1011 corresponding to the TMCP part. Fragmentation of the metabolite formed by oxidative cleavage of the morpholine ring (MB2) with PI at *m/z* 286.1801 resulted in a specific FI at *m/z* 188.0706 which occurred from a split of the TMCP moiety. As above mentioned for JWH-200, FI at *m/z* 112.0756 in the HRMS^2^ spectrum of dehydro-A-796260 (MB8) with PI at *m/z* 353.2223 indicated a previous hydroxylation either on the morpholine ring or ethyl spacer with subsequent water elimination. Besides, the parallel occurrence of FI at *m/z* 98.0607 varying from FI at *m/z* 114.0913 by one methyl group (− 16.0313 u) suggested that the dehydrogenation happened at the morpholine ring. This finding was in line with dehydrogenated JWH-200 metabolite described by De Brabanter et al. (2013). Two possible metabolic reactions must be considered regarding MB11 with PI at *m/z* 369.2172. Either it involved an aldehyde formation at the TMCP moiety or a dihydroxylation and subsequent dehydrogenation resulting in a cyclization as postulated for the structural related compound XLR-11 in human hepatocytes (Wohlfarth et al. 2013). Both formations could be justified by FI at *m/z* 111.0804 deriving from FI at *m/z* 97.1011 by one more oxygen and two less hydrogen atoms. However, as aldehydes are highly reactive and might be altered before detection, the more likely structure of MB11 is given in Fig. 7. MB15 with PI at *m/z* 371.2329 represented one of three metabolites with the hydroxy group located at the TMCP ring or vicinal carbonyl (MB14–MB16). Hence, the following reaction positions were possible for a hydroxylation: one of the methyl substituents, the alpha carbon to the carbonyl group and the carbonyl carbon leading to a hemiketal formation as described by Wohlfarth et al. (2013). Regarding MB15, it was presumed to be hydroxylated at the alpha carbon to the carbonyl group, firstly due to FI at *m/z* 353.2223, which indicated a loss of water. This led to a putative opening of the cyclopropyl ring. Second, the FI at *m/z* 313.1910 suggested an elimination of C_3_H_4_ (− 40.0302 u) from the opened TMCP ring, which was specific for MB15.

### 5F-EMB-PINACA

The fragmentation pattern of 5F-EMB-PINACA with PI at *m/z* 378.2187 showed an initial ethanol loss due to an ester cleavage by FI at *m/z* 332.1775. Cleavage at the carbonyl carbon of the ester moiety resulted in in a loss of CO (− 27.9949 u) representing by FI at *m/z* 304.1825. On that basis, FI at *m/z* 233.1084 was formed by breaking of the amide bond between the carbonyl carbon and the nitrogen atom. The FI at *m/z* 213.1022 derived therefrom by cleavage of the hydrogen fluoride (HF) at the pentyl side chain. A different mechanism was assumed for the formation of FI at *m/z* 251.1190, which was shifted by a mass of + 18.0106 u compared to FI at *m/z* 233.1084, which corresponded to the mass of water. As discussed for the methyl ester homologs 5F-ADB and 4F-MDMB-BINACA (Richter et al. 2019b; Wagmann et al. 2020) a rearrangement reaction by ester cleavage and subsequent nucleophilic attack of the oxygen at the indazole nitrogen atom in the second position was most likely the cause. The same mass shift was observed for FIs at *m/z* 145.0396 and 163.0502 consisting of the vicinal carbonyl group to the indazole moiety coupled to the fluoro pentyl chain. A fluorine rearrangement was supposed to be involved in the emergence of FI at *m/z* 177.0458, which was shifted by—C_4_H_8_ from FI at *m/z* 233.1084. Diao et al. identified exact the same fragment in the spectra of derivates with a pentyl fluoride side chain such as FUBIMINA and THJ-2201 and postulated the formation of an acyl fluoride without exact mechanism. Furthermore, the cleavage between alpha and beta carbon atom to the indazole led to a butenyl loss (Diao et al. 2016). Finally, FI at *m/z* 69.0698 could be explained by the pentenyl side chain after loss of the indazole part and HF.

One metabolite generated by oxidative defluorination together with ester hydrolysis (MC5) with PI at *m/z* 348.1917 was identified due to the absence of the fragment originating from an ester hydrolysis by mass shift of 28.0313 u from the parent mass, which was present in all spectra of metabolites with an intact ester group. In addition, FI at *m/z* 302.1863 differed from FI at m/z 304.1825 by one more oxygen and one less fluorine atom. MC6 with a PI at *m/z* 348.1717 was suspected to be formed after ester hydrolysis by a cyclization to a lactone as postulated for 4F-MDMB-BINACA by Wagmann et al. (2020). Although an ester hydrolysis followed by dehydrogenation would be also possible (Haschimi et al. 2019; Krotulski et al. 2019), the fact that FI at *m/z* 320.1769 was present in the spectra of both MC6 as well as MC10 (ester hydrolysis + hydroxylation at the isopropyl part), but not in that of the ester hydrolysis (MC7) supports the assumption of a lactone formation. Similar to 4F-MDMB-BINACA, FI at *m/z* 99.0440 corresponded to the lactone part after cleavage between alpha carbon and amide nitrogen atom (Wagmann et al. 2020). The aforementioned ester hydrolysis product with PI at *m/z* 350.1874 was identified by the FI at *m/z* 304.1825, which was already stated for the parent mass. MC10 with PI at *m/z* 366.1823 originated, as above mentioned from an ester hydrolysis and hydroxylation at the isopropyl part. The unaltered FI at *m/z* 251.1190 compared to the parent spectrum ruled out a hydroxylation at the indazole or fluoro pentyl part. Besides, the mass shift of -18.0106 u leading to 348.1717 indicated a water loss, which pointed out that the hydroxylation happened at the isopropyl part.

### Isozyme mapping

The initial metabolic steps of all three SC were primarily catalyzed by CYP3A4 and CYP3A5, but various other isozymes were additionally involved. In case of 5F-EMB-PINACA, due to the presence of human carboxylesterases only in pHLM, the ester hydrolysis (MC7) was exclusively found in pHLM incubations but not during the isozyme mapping. All other initial metabolites were only detected in pHLM incubations in combination with the ester hydrolysis (data not shown), thus, an esterase activity screening of 5F-EMB-PINACA might give further insights (Meyer et al. 2015; Wagmann et al. 2020). As the extensive phase I metabolism is mediated by several isozymes, drug–drug or drug–food interaction are unlikely.

## Conclusion

With a successfully optimized HCSA method, the cytotoxic potential of the SC JWH-200, A-796260, and 5F-EMB-PINACA was evaluated. The naphthylindole JWH-200 and tetramethylcyclopropanoylindole A-796260 showed the strongest cytotoxic effects, whereas the potential of indazole carboxamide 5F-EMB-PINACA was moderate. All SC caused a collapse of the mitochondrial membrane potential, which was found to be the most reliable/sensitive indicator. No metabolism-related impact was observed; however, the low enzymatic activity of HepG2 cells could be a limitation. Several phase I metabolites of the studied SC could be identified in pHLM or single isozyme incubations. Most abundant metabolites of JWH-200 in pHLM were formed by *N*-dealkylation, oxidative morpholine cleavage, and oxidative morpholine opening. Oxidative morpholine cleavage, oxidative morpholine opening, hydroxylation, and dihydroxylation followed by dehydrogenation were the most abundant metabolites of A-796260. 5F-EMB-PINACA underwent mainly ester hydrolysis plus additional metabolic steps such as oxidative defluorination, oxidative defluorination to carboxylic acid, and hydroxylation. The initial metabolic steps of all three SC were primarily catalyzed by CYP3A4 and CYP3A5 but several other isozymes were involved making interaction unlikely.

## Supplementary Information

Below is the link to the electronic supplementary material.

Supplementary file1 (PDF 3329 kb)
