# Supplementary material for: Cytotoxicity, metabolism, and isozyme mapping of the synthetic cannabinoids JWH-200, A-796260, and 5F-EMB-PINACA studied by means of in vitro systems
Source: Arch Toxicol. 2021 Aug 28;95(11):3539–57. doi: 10.1007/s00204-021-03148-3 (PMC8492589; doi:10.1007/s00204-021-03148-3)
Supplement: Supplementary file 1 — Supplementary file1 (PDF 3329 kb) [file 204_2021_3148_MOESM1_ESM.pdf]

Archives of Toxicology

Electronic Supplementary Material

Cytotoxicity, metabolism, and isozyme mapping of the synthetic cannabinoids JWH-200, A-796260, and 5F-EMB-PINACA studied by means of in vitro systems

Tanja M. Gampfer, Lea Wagmann, Anouar Belkacemi, Veit Flockerzi, Markus R. Meyer

Isozyme mapping and in vitro phase I metabolism

JWH-200, A-796269, or 5F-EMB-PINACA were incubated at a final concentration of 25 µM with 50 pmol/ml of CYP1A2, CYP2A6, CYP2B6, CYP2C8, CYP2C9, CYP2C19, CYP2D6, CYP2E1, CYP3A4, and CYP3A5, respectively, 0.25 mg protein/mL FMO3, or 1 mg microsomal protein/mL pHLM. Furthermore, the incubation mixtures contained the following components: isocitrate (5 mM), isocitrate dehydrogenase (0.5 U/mL), $MgCl_2$ (5 mM), $NADP^+$ (1.2 mM), 90 mM phosphate buffer (pH 7.4), and superoxide dismutase (200 U/mL). As recommended by the manufacturer, incubations with CYP2A6 and CYP2C9 were conducted by replacing phosphate buffer with Tris buffer. Incubations (50 µL final volume) were performed at 37°C for 30 min and terminated by adding 50 µL of ice-cold acetonitrile. Afterwards, the mixtures were centrifuged at 18,407 × *g* for 5 min, the supernatants were transferred into autosampler vials, and 1 µL was injected onto the LC-HRMS/MS system. Incubations with pHLM were used as positive controls. Negative controls (without enzyme) were additionally prepared to identify non-metabolically formed compounds. All incubations were done in duplicate.

LC-HRMS/MS apparatus for identification of metabolites

All samples were analyzed using an Orbitrap-based ThermoFisher Scientific (TF, Dreieich, Germany) Q-Exactive Plus equipped with a heated electrospray ionization source (HESI)-II source, which was coupled to a TF Dionex UltiMate 3000 rapid separation pump assembled with a degasser, a quaternary pump, and an UltiMate autosampler. An external mass calibration was done prior to analysis. Gradient elution was performed on a TF Accucore PhenylHexyl column (100 mm x 2.1 mm, 2.6 µm). The mobile phase was composed of 2 mM aqueous ammonium formate containing formic acid (0.1%, v/v, pH 3, eluent A) and 2 mM ammonium formate solution in acetonitrile:methanol (1:1, v/v), water (1%, v/v), and formic acid (0.1%, v/v, eluent B). Initially, the flow rate was set at 500 µL/min for 10 min followed by 800 µL for 10–13.5 min. The following stepped gradient was used: from 0–1 min hold 99% A, 1–10 min to 1% A, 10–11.5 min hold 1% A, and 11.5–13.5 min hold 99% A. HESI-II source parameters were set as follows: heater temperature, 320°C; ion transfer capillary temperature, 320°C; spray voltage, 4.0 kV; ionization mode, positive; sheath gas, 60 arbitrary units (AU); auxiliary gas, 10 AU; sweep gas, 0 AU; and S-lens RF level, 50.0. Parent compounds and metabolites were identified by high-resolution (HR) full scan mode and subsequent targeted $HRMS^2$ mode using an inclusion list, which was composed of the exact masses of the respective parent compound and its hypothetical metabolites. Full scan data acquisition was performed as follows: resolution, 35,000 at mass-to-charge ratio (*m/z*) 200; microscans, 1; automatic gain control (AGC) target, $1e^6$; maximum injection time (mIT), 120 ms; and scan range, at *m/z* 50 – 750. The settings of the targeted $HRMS^2$ mode were as follows: option "pick others", enabled; dynamic exclusion, disabled; resolution, 17,500 at *m/z* 200; microscans, 1; isolation window, 1.0 *m/z*; loop count, 5; AGC target, $2e^5$; mIT, 250 ms; high collision dissociation cell with stepped normalized collision energy, 17.5, 35.0, 52.5; exclude isotopes, on; spectrum data type,

profile; and underfill ratio, 1%. ChemSketch 2010 12.01 (ACD/Labs, Toronto, Canada) was used for chemical structure drawings of hypothetical metabolites and exact mass calculations. A maximal mass difference between calculated and measured mass of 5 ppm in the HR full scan was defined as acceptable. TF Xcalibur Qual Browser software version 2.2 was used for data handling.

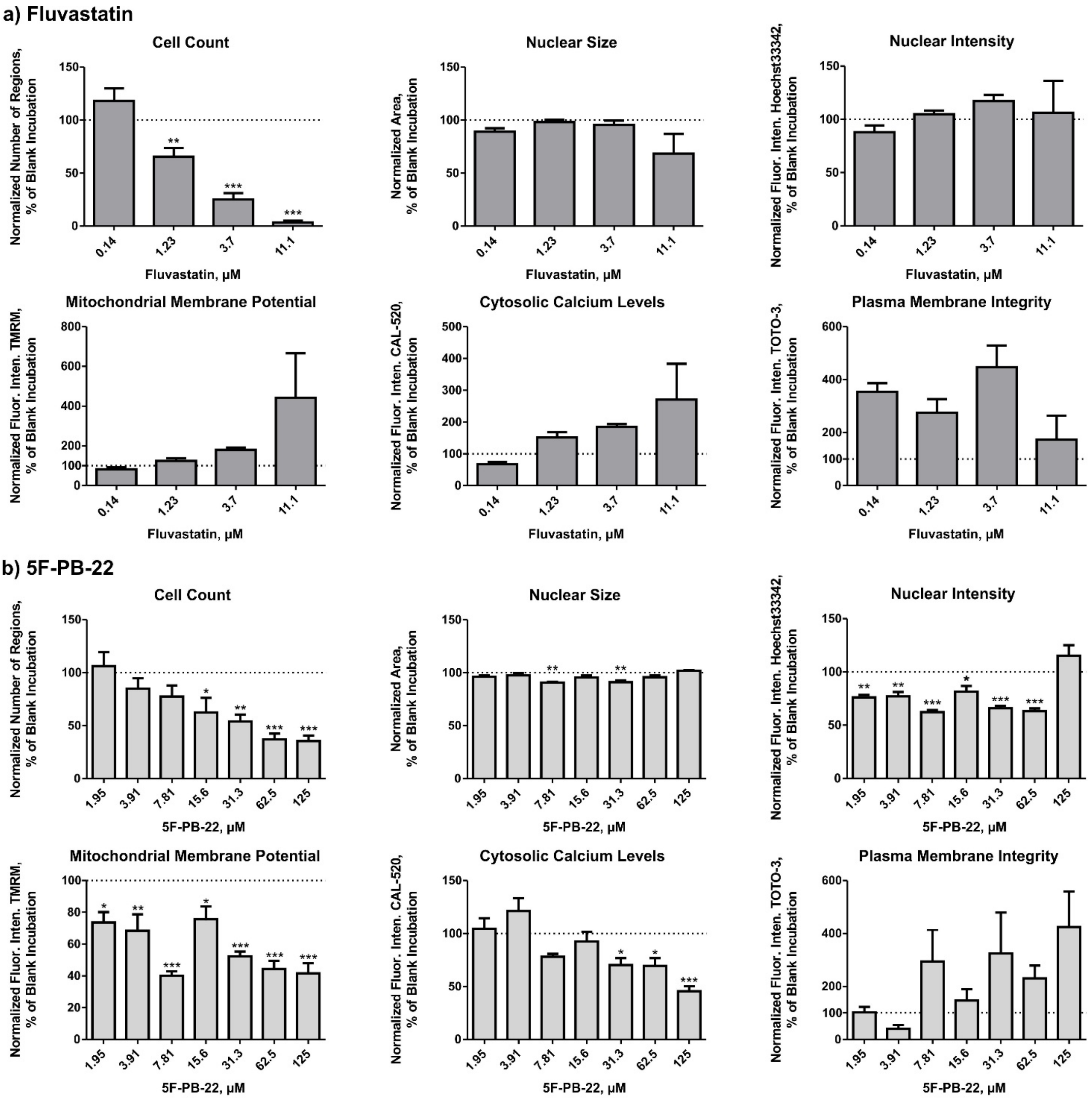

Figure S1 Dose-response plots of fluvastatin (a) and 5F-PB-22 (b) obtained by incubation of different concentrations (0.14, 1.23, 3.7 and 11.1 µM, fluvastatin; 1.95, 3.91, 7.81, 15.6, 31.3, 62.5, and 125 µM, 5F-PB-22) and blank incubations without test compound. Changes on different parameters (cell count, nuclear size, nuclear intensity, mitochondrial membrane potential, cytosolic calcium levels, and plasma membrane integrity) are plotted in relation to blank incubations (100%). All parameters were normalized to the cell count except the cell count, which was normalized to the number of appropriate images. Values are expressed as mean ± standard error of the mean (SEM; n = 5). Statistical analysis was done using one-way ANOVA followed by Dunnett's post-hoc test (***, $P < 0.001$, **, $P < 0.01$, *, $P < 0.05$ compared to blank incubation). Fluor. Inten., Fluorescence intensity.

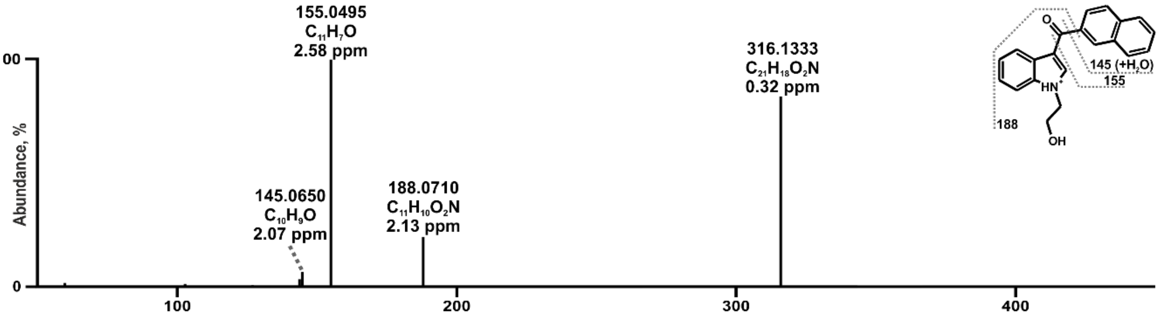

MA2 oxidative morpholine cleavage, MS^2 at *m/z* 316.1332, RT 7.08 min

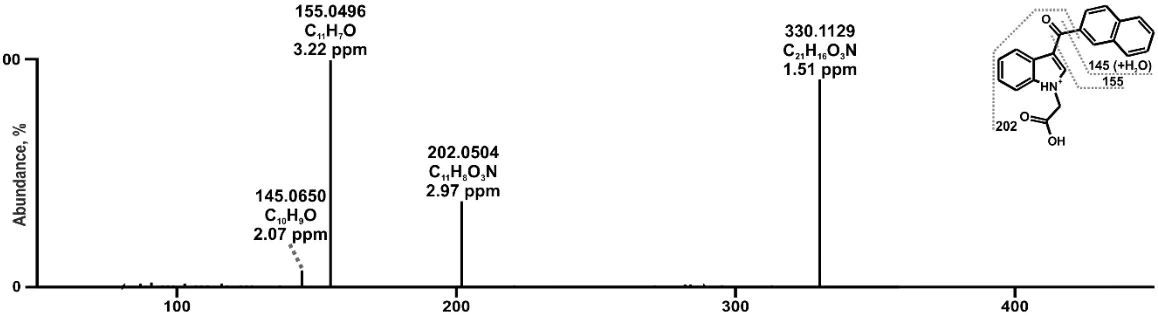

MA3 oxidative morpholine cleavage + oxidation (COOH), MS^2 at *m/z* 330.1124, RT 7.02 min

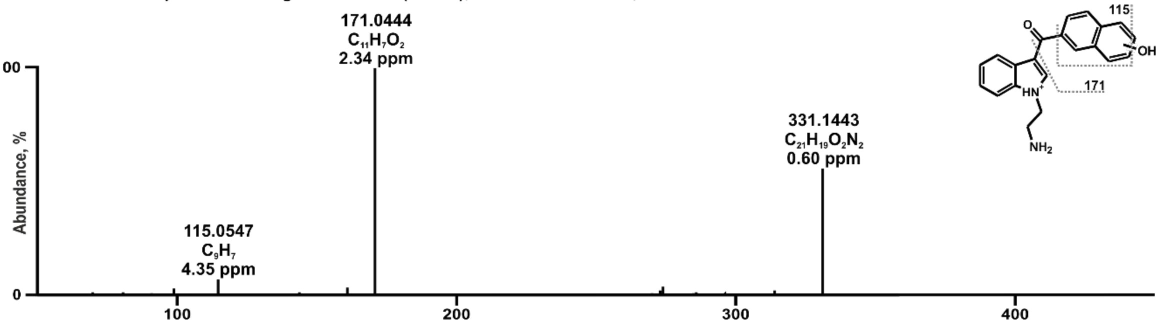

MA4 *N*-dealkylation + hydroxylation at the naphthalene, MS^2 at *m/z* 331.1441, RT 5.05 min

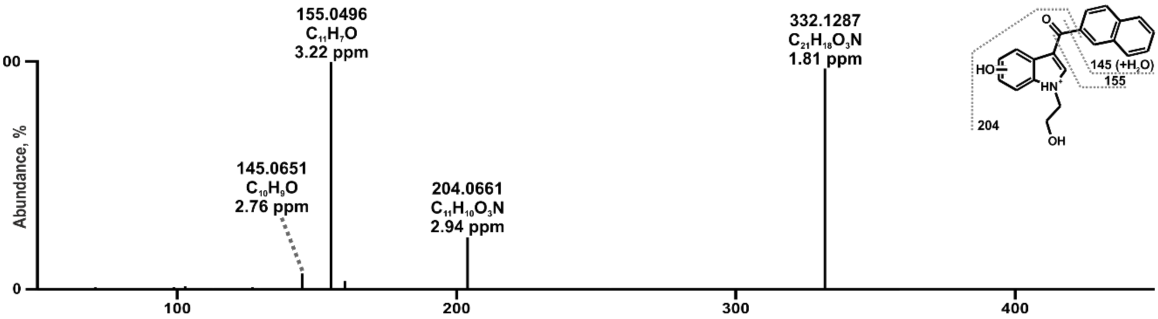

MA5 oxidative morpholine cleavage + hydroxylation at the indole isomer 1, MS^2 at *m/z* 332.1281, RT 6.20 min

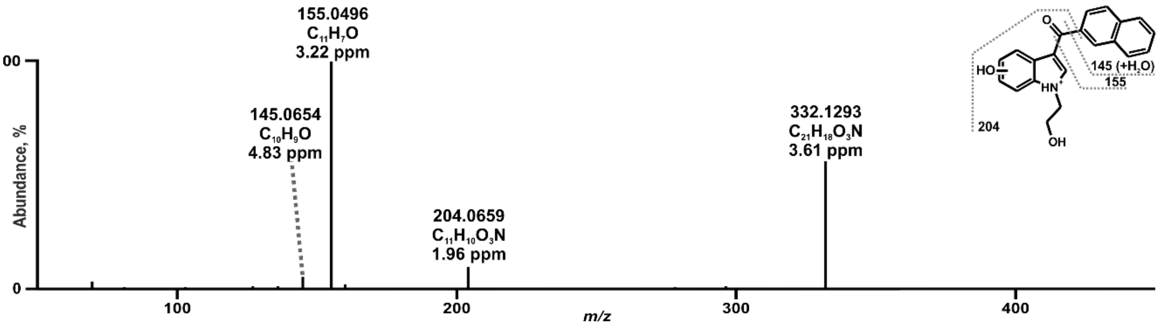

MA6 oxidative morpholine cleavage + hydroxylation at the indole isomer 2, MS^2 at *m/z* 332.1281, RT 6.34 min

Figure S2 HRMS^2 spectra of the remaining JWH-200 metabolites identified in pooled human liver microsomes or isozyme incubations. Metabolites are ordered by increasing mass and retention time (RT). Metabolite-IDs correspond to Table S1. JWH-200 metabolite (MA). Metabolites with an asterisk are regarded as artifacts.

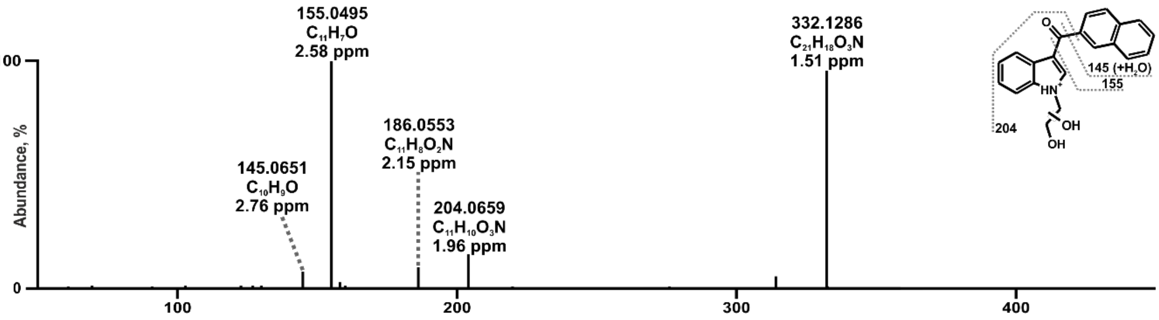

MA7 oxidative morpholine cleavage + hydroxylation at the ethyl part isomer 3, MS^2 at *m/z* 332.1281, RT 6.74 min

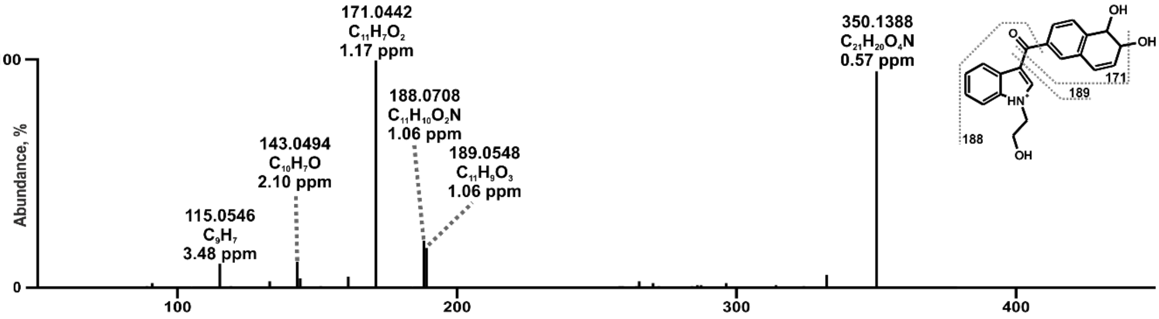

MA8 oxidative morpholine cleavage + epoxidation + non-enzymatic hydrolysis, MS^2 at *m/z* 350.1386, RT 5.33 min

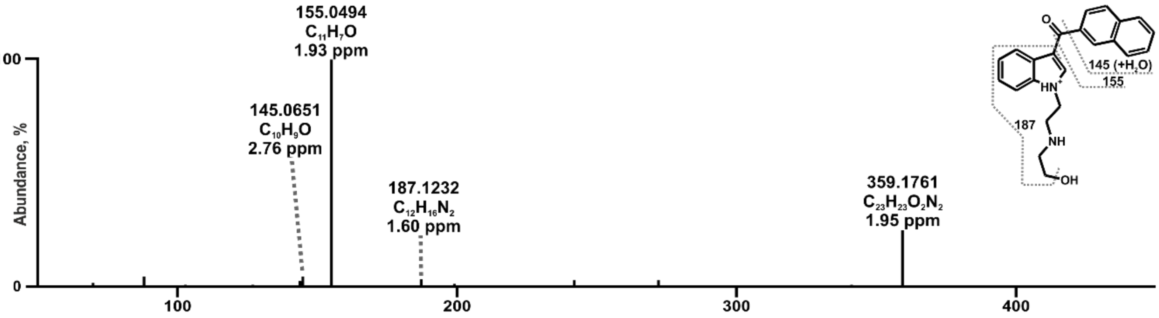

MA9 oxidative morpholine ring opening isomer 1, MS^2 at *m/z* 359.1754, RT 5.67 min

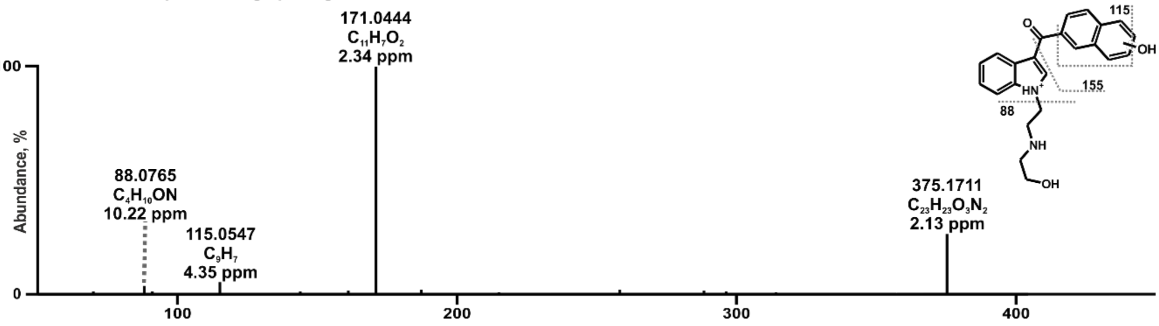

MA11 oxidative morpholine ring opening + hydroxylation at the naphthalene isomer 2, MS^2 at *m/z* 375.1703, RT 5.08 min

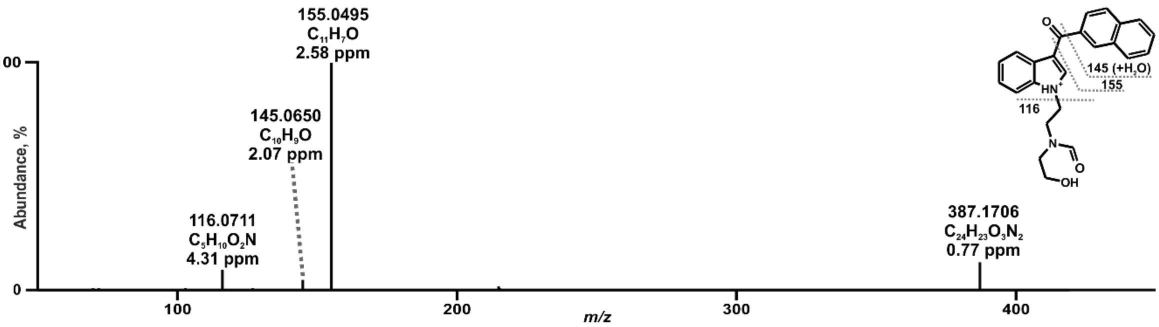

MA12 oxidative morpholine ring opening isomer 2, MS^2 at *m/z* 387.1703, RT 6.66 min

Figure S2 continued.

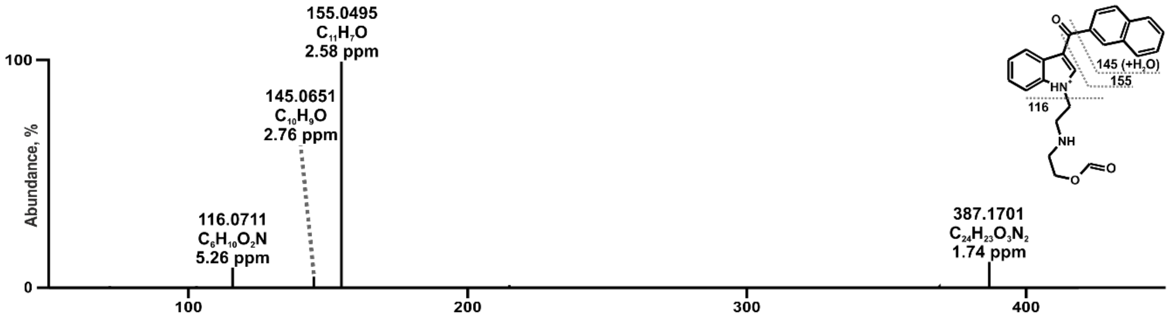

MA13 oxidative morpholine ring opening isomer 3, MS^2 at *m/z* 387.1703, RT 6.75 min

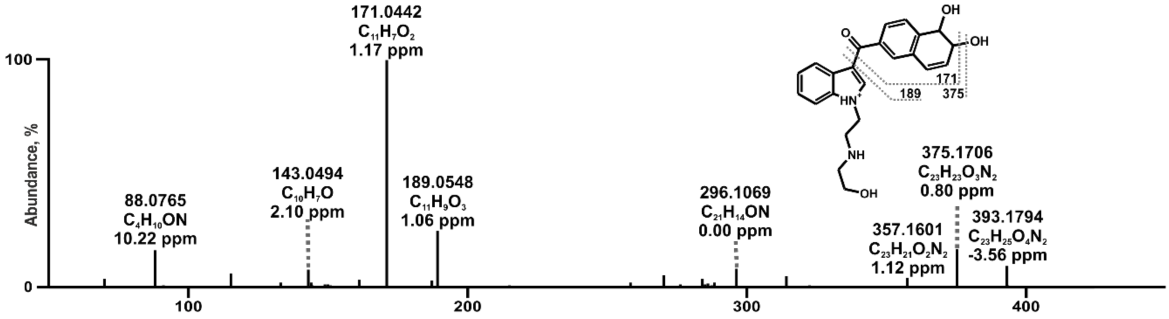

MA14 oxidative morpholine ring opening + epoxidation + non-enzymatic hydrolysis, MS^2 at *m/z* 393.1808, RT 4.09 min

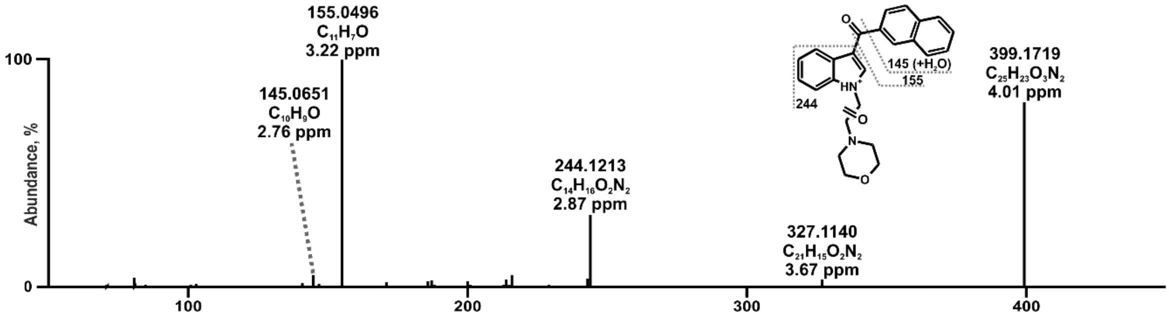

MA15 hydroxylation at the ethyl part + oxidation isomer 1, MS^2 at *m/z* 399.1703, RT 5.60 min

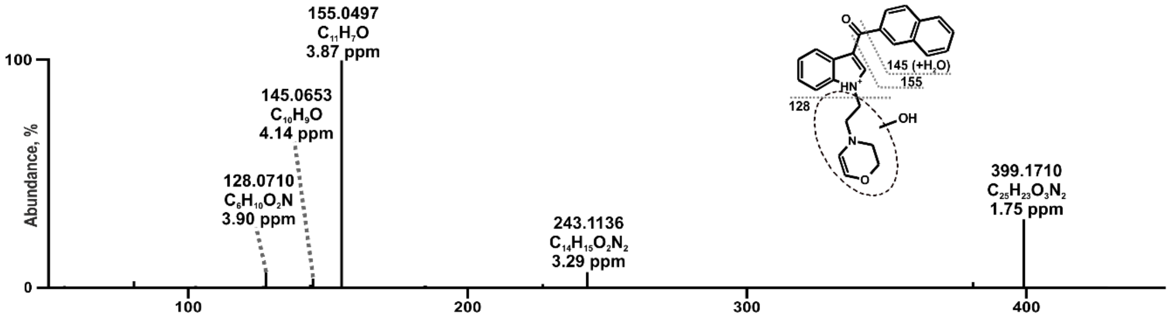

MA16 dihydroxylation at the morpholine + dehydrogenation, MS^2 at *m/z* 399.1703, RT 6.96 min

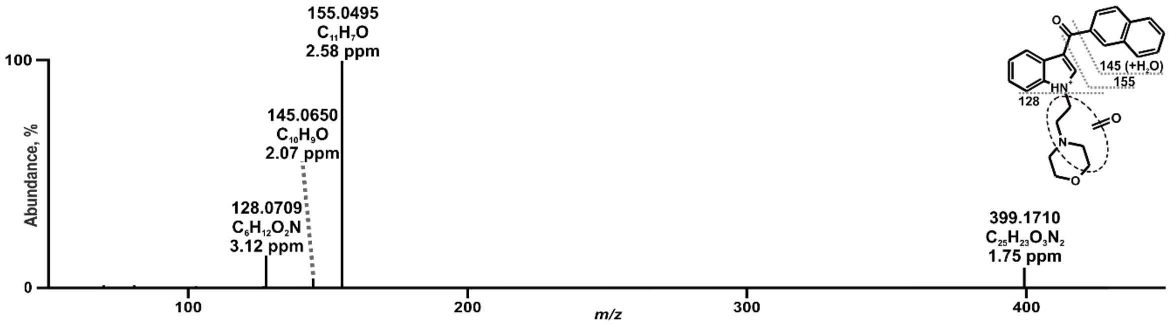

MA17 hydroxylation at the morpholine or ethyl part + oxidation isomer 2, MS^2 at *m/z* 399.1703, RT 7.18 min

Figure S2 continued.

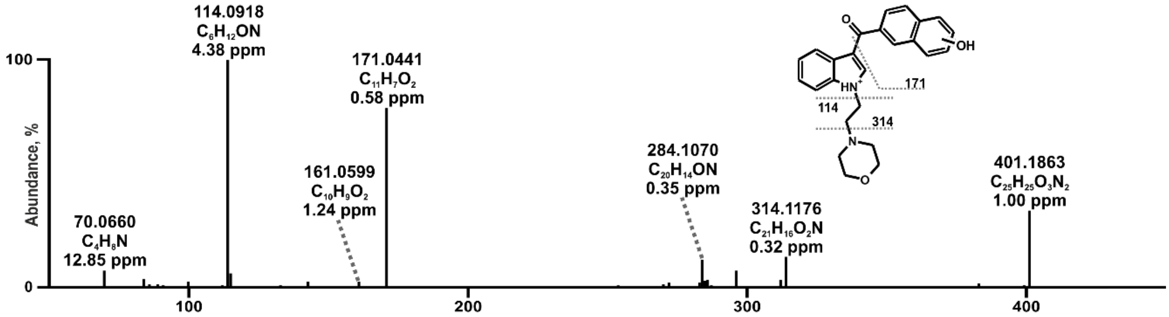

MA18 hydroxylation at the naphthalene isomer 1, MS[2] at *m/z* 401.1859, RT 5.44 min

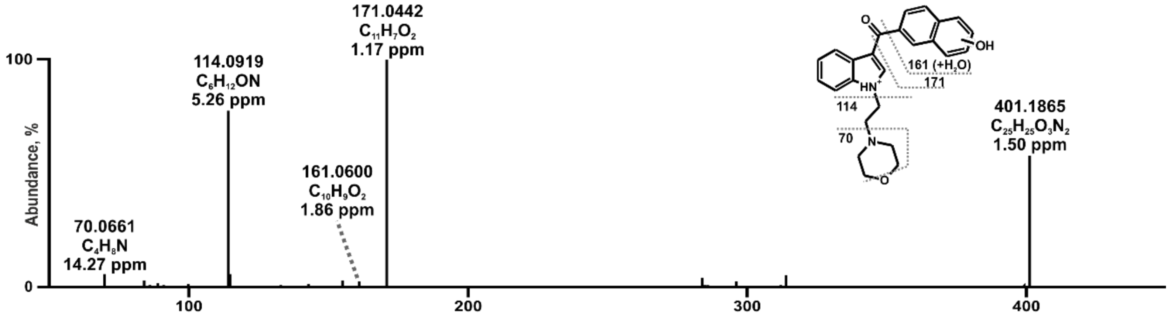

MA19 hydroxylation at the naphthalene isomer 2, MS[2] at *m/z* 401.1859, RT 5.53 min

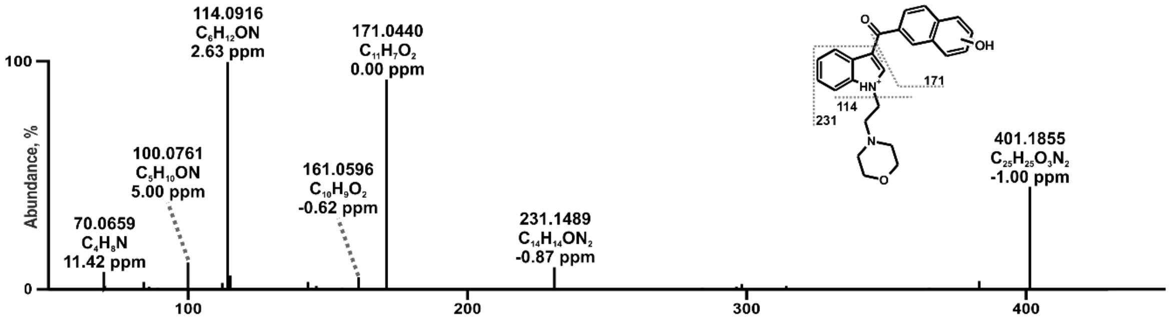

MA20 hydroxylation at the naphthalene isomer 3, MS[2] at *m/z* 401.1859, RT 5.62 min

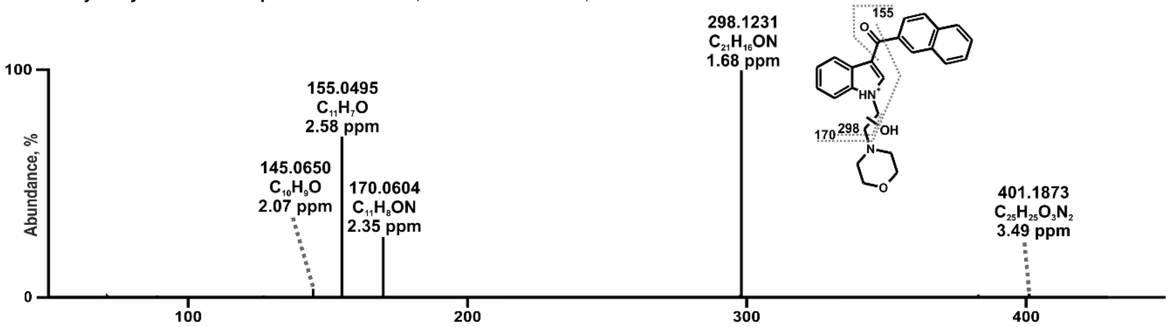

MA21* hydroxylation at the ethyl part, MS[2] at *m/z* 401.1859, RT 6.09 min

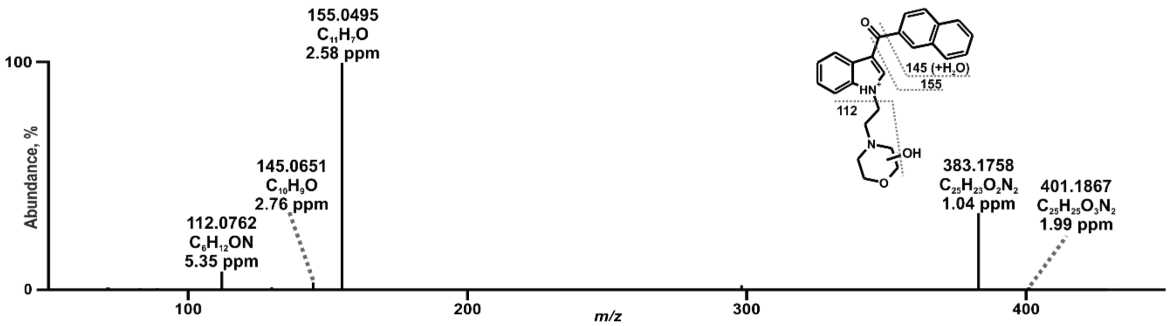

MA22 hydroxylation at the morpholine isomer 1, MS[2] at *m/z* 401.1859, RT 6.26 min

Figure S2 continued.

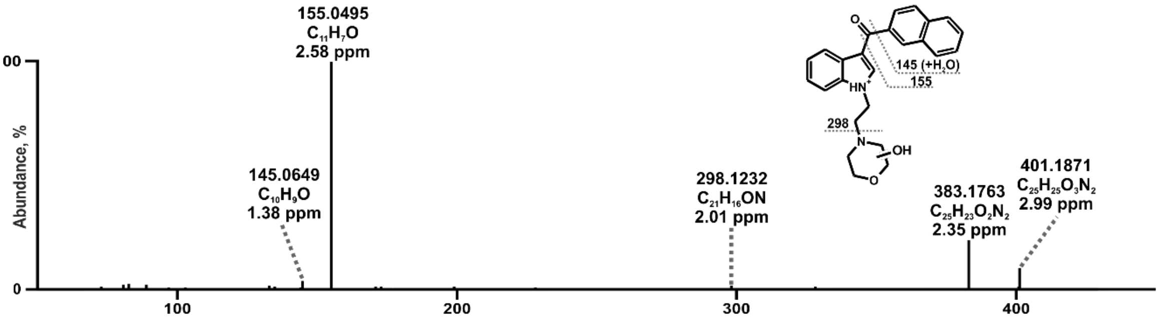

MA25 hydroxylation at the morpholine isomer 2, MS^2 at *m/z* 401.1859, RT 6.70 min

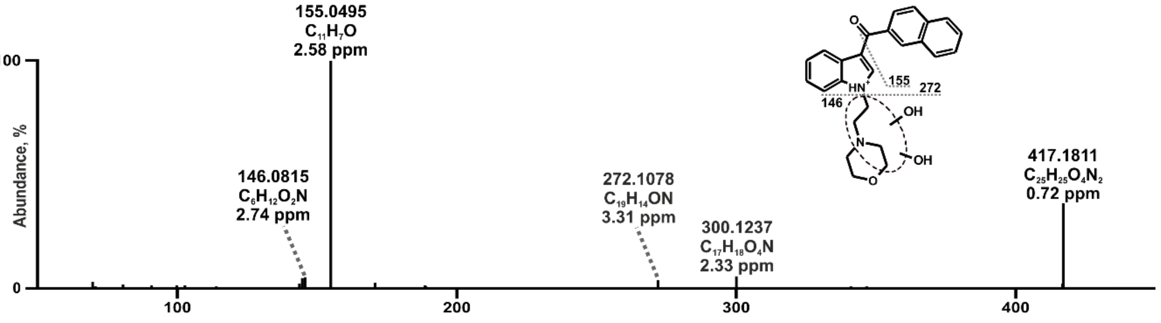

MA26 dihydroxylation at the morpholine or ethyl part, MS^2 at *m/z* 417.1808, RT 5.90 min

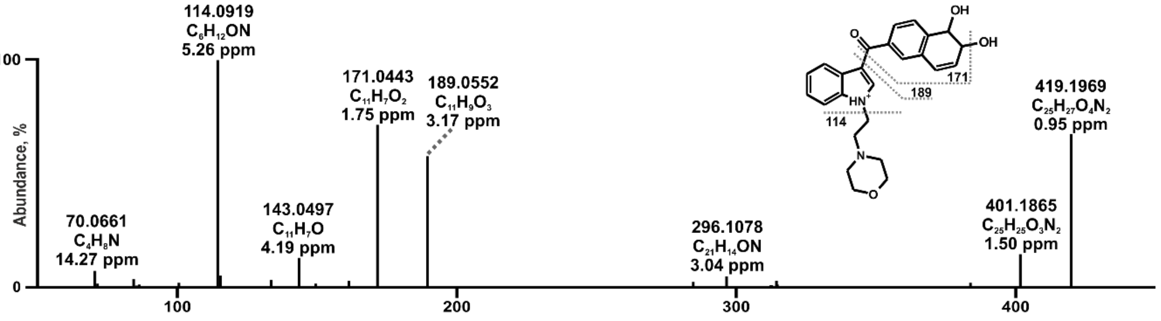

MA27 epoxidation + non-enzymatic hydrolysis isomer 1, MS^2 at *m/z* 419.1965, RT 4.44 min

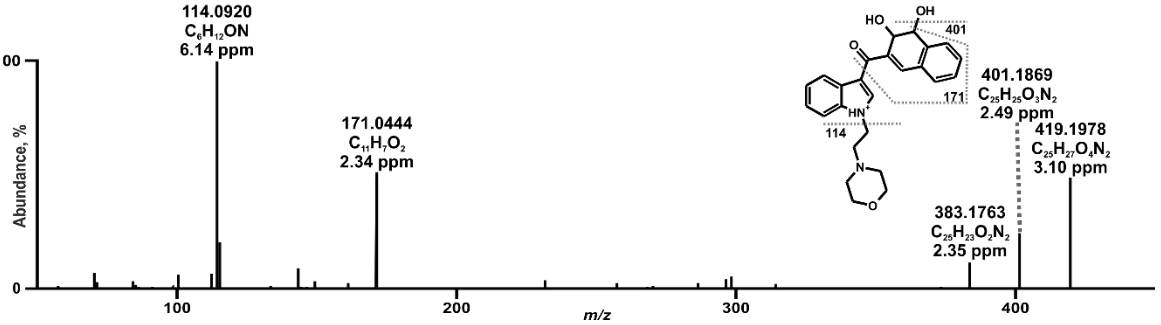

MA28 epoxidation + non-enzymatic hydrolysis isomer 2, MS^2 at *m/z* 419.1965, RT 4.86 min

Figure S2 continued.

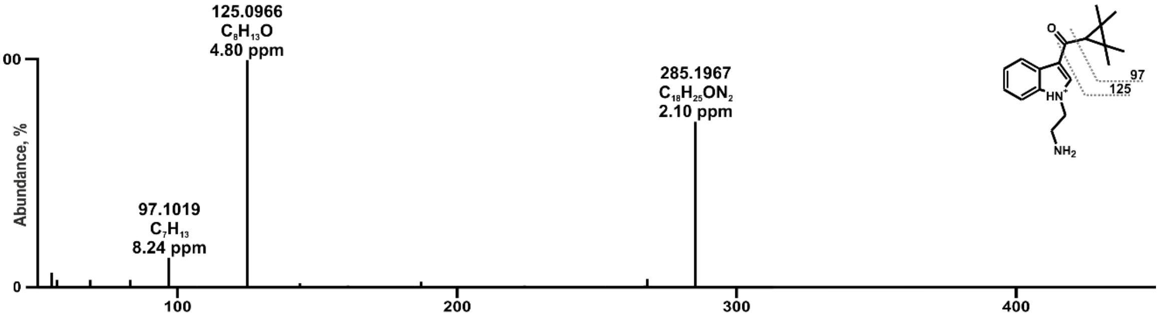

MB1 *N*-dealkylation, MS^2 at *m/z* 285.1961, RT 5.91 min

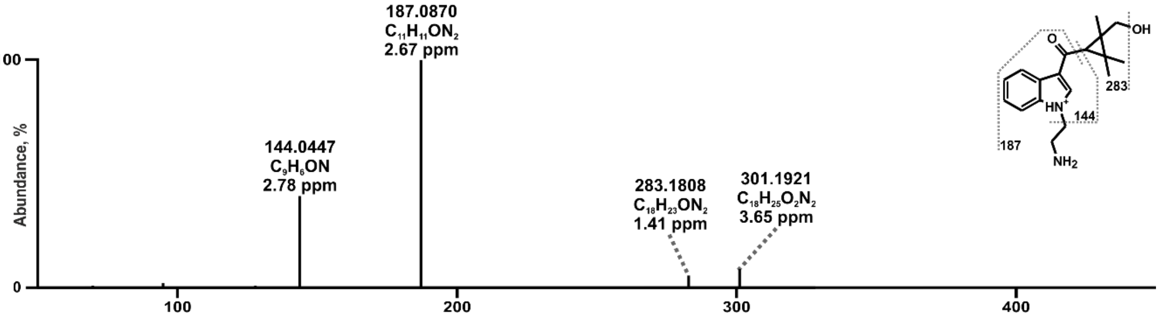

MB3 *N*-dealkylation + hydroxylation at the TMCP part, MS^2 at *m/z* 301.1910, RT 4.61 min

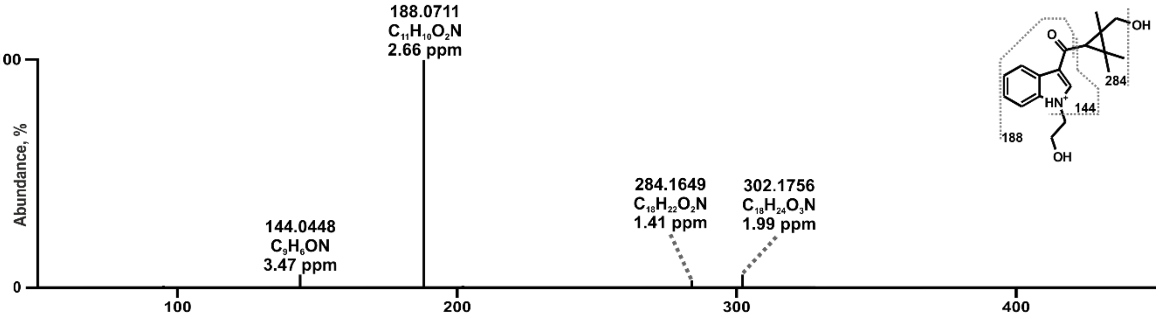

MB4 oxidative morpholine ring cleavage + hydroxylation at the TMCP part, MS^2 at *m/z* 302.1750, RT 6.05 min

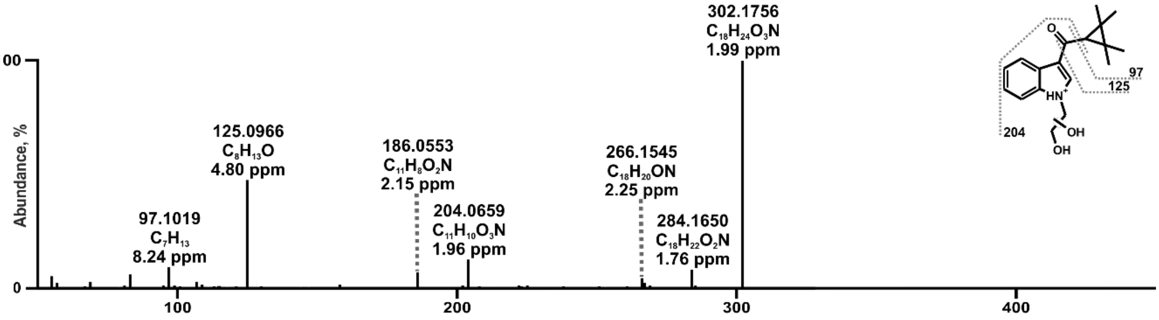

MB5 oxidative morpholine ring cleavage + hydroxylation at the ethyl part, MS^2 at *m/z* 302.1750, RT 7.01 min

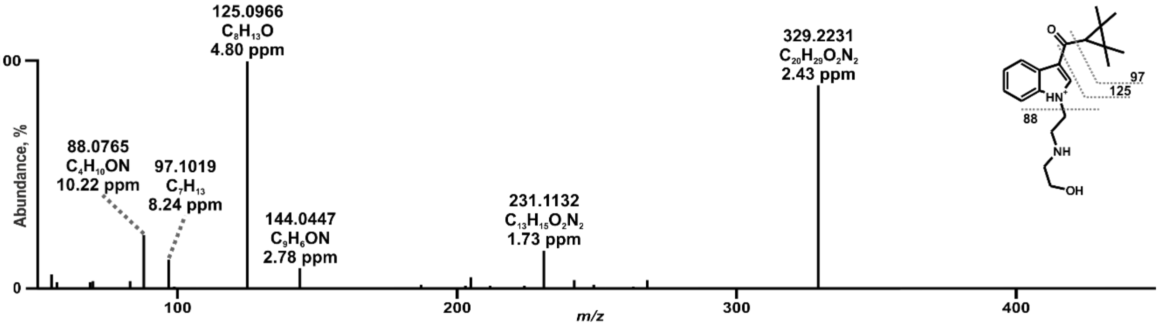

MB6 oxidative morpholine ring opening isomer 1, MS^2 at *m/z* 329.2223, RT 5.93 min

Figure S3 HRMS^2 spectra of the remaining A-796260 metabolites identified in pooled human liver microsomes or isozyme incubations. Metabolites are ordered by increasing mass and retention time (RT). Metabolite-IDs correspond to Table S2. A-796260 metabolite (MB). Metabolites with an asterisk are regarded as artifacts.

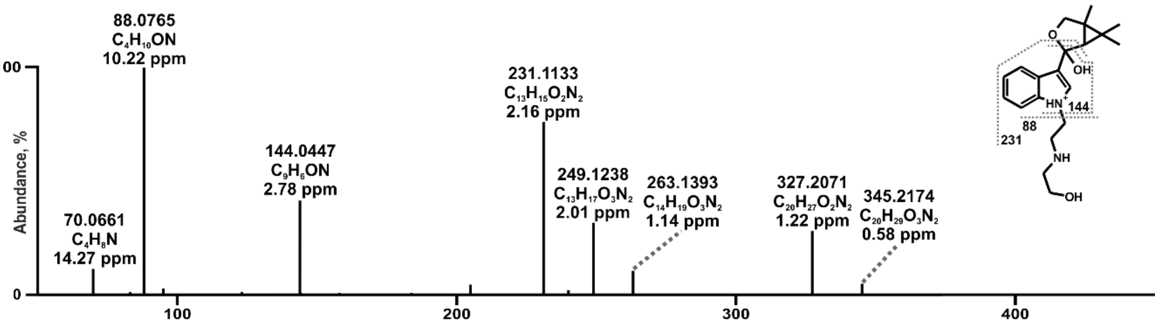

MB7 oxidative morpholine ring opening + hydroxylation at the TMCP part isomer 1 (hemiketal formation), MS^2 at *m/z* 345.2172, RT 4.62 min

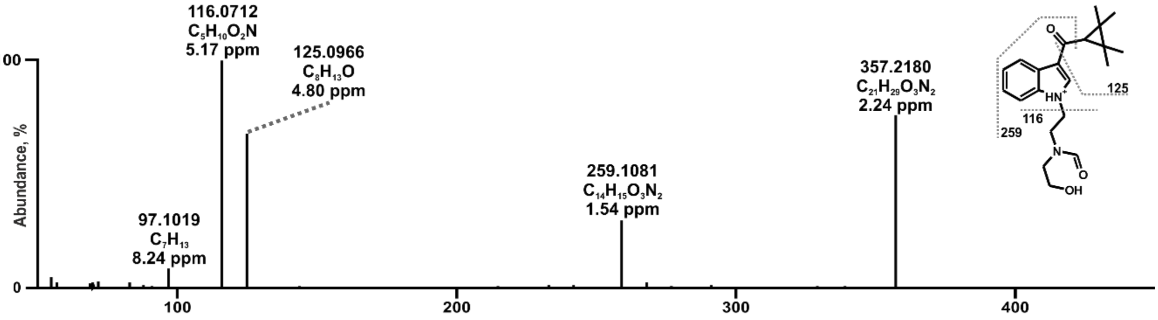

MB9 oxidative morpholine ring opening isomer 2, MS^2 at *m/z* 357.2172, RT 6.94 min

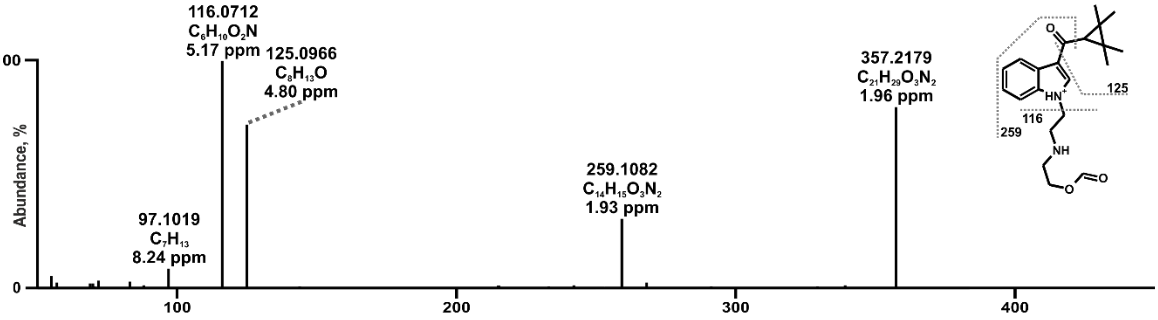

MB10 oxidative morpholine ring opening isomer 3, MS^2 at *m/z* 357.2172, RT 7.08 min

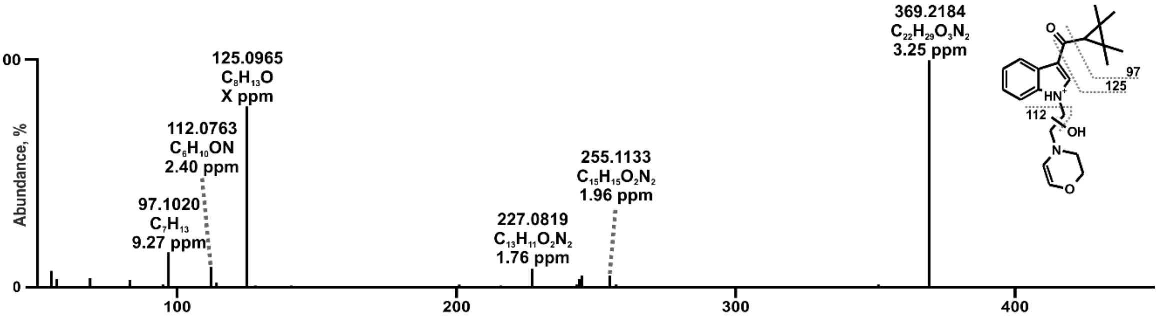

MB12 dihydroxylation at the morpholine and ethyl part + dehydrogenation, MS^2 at *m/z* 369.2172, RT 5.95 min

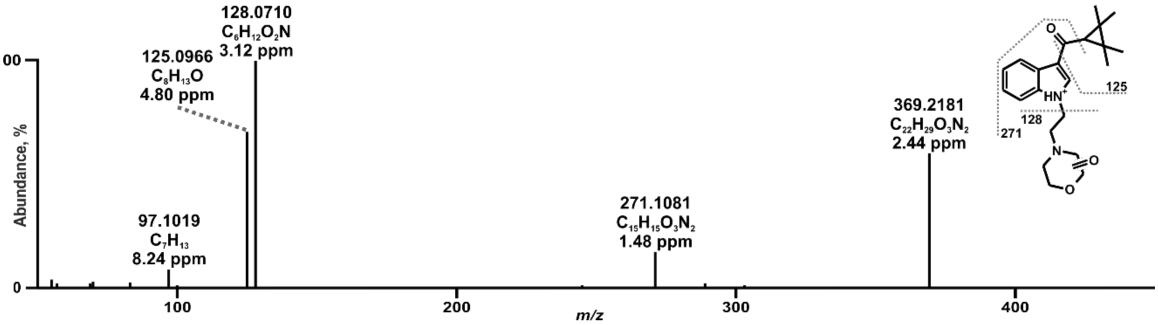

MB13 hydroxylation at the morpholine + oxidation, MS^2 at *m/z* 369.2172, RT 7.53 min

Figure S3 continued.

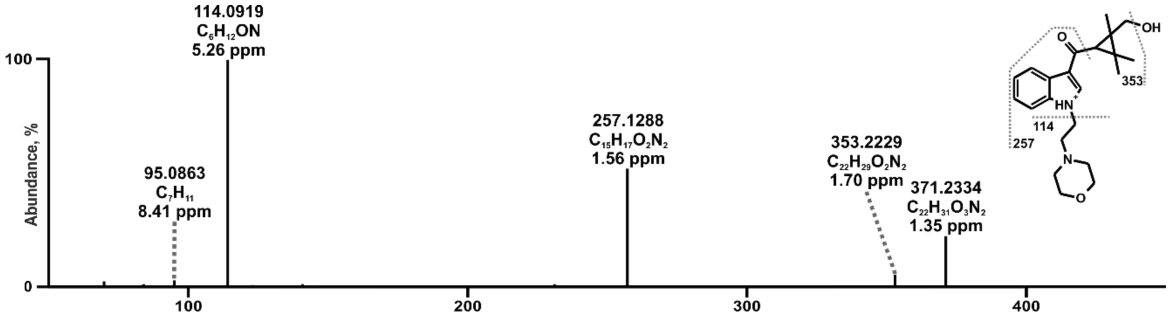

MB14 hydroxylation at the TMCP part isomer 1, MS^2 at *m/z* 371.2329, RT 4.95 min

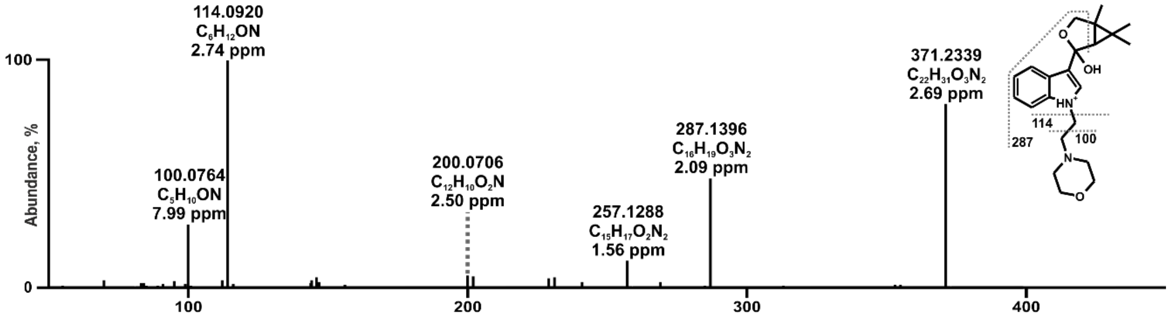

MB16 hydroxylation at the TMCP part isomer 3 (hemiketal formation), MS^2 at *m/z* 371.2329, RT 5.66 min

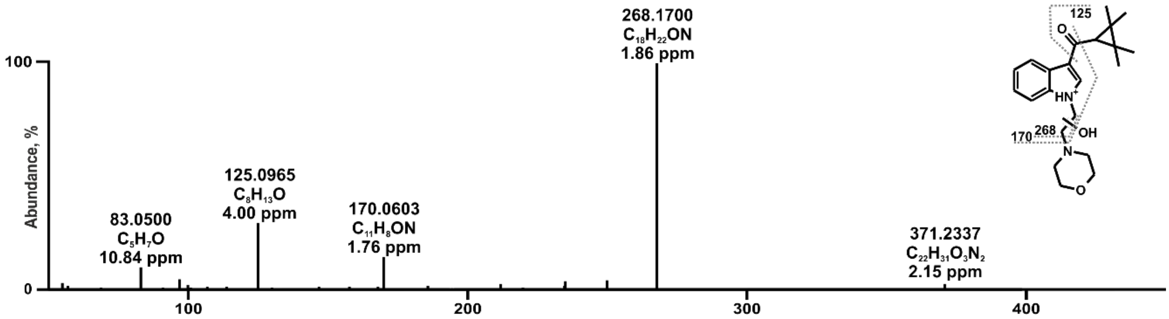

MB17* hydroxylation at the ethyl part, MS^2 at *m/z* 371.2329, RT 6.35 min

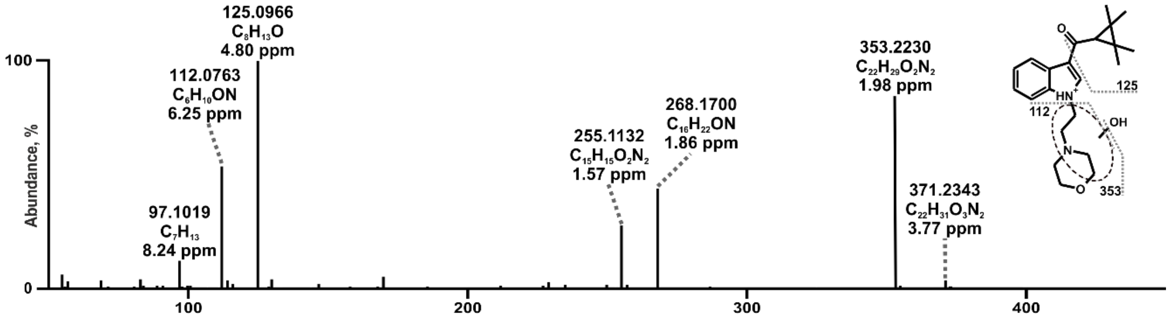

MB18 hydroxylation at the morpholine or ethyl part, MS^2 at *m/z* 371.2329, RT 6.42 min

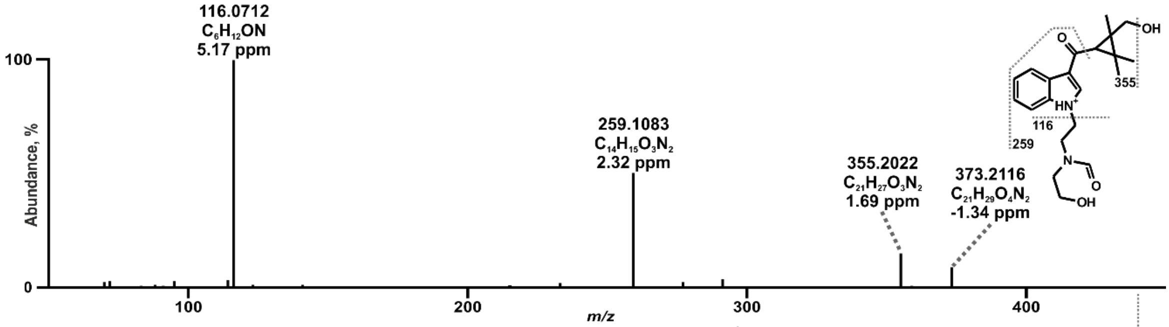

MB19 oxidative morpholine ring opening + hydroxylation at the TMCP part isomer 2, MS^2 at *m/z* 373.2121, RT 5.61 min

Figure S3 continued.

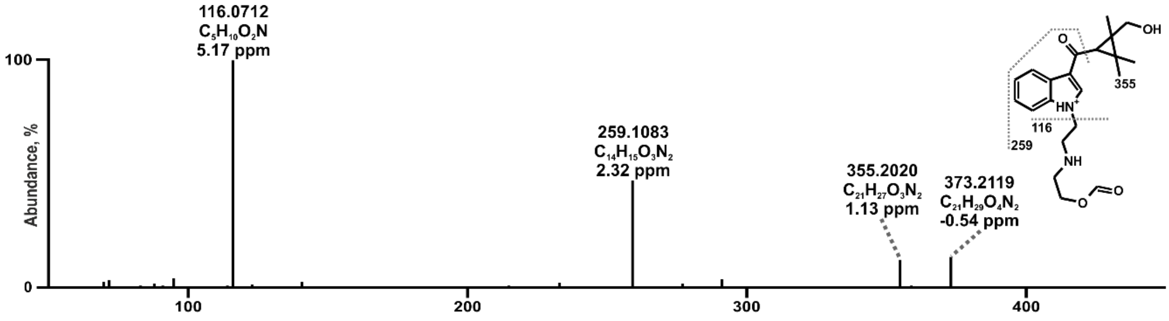

MB20 oxidative morpholine ring opening + hydroxylation at the TMCP part isomer 3, MS^2 at *m/z* 373.2121, RT 5.76 min

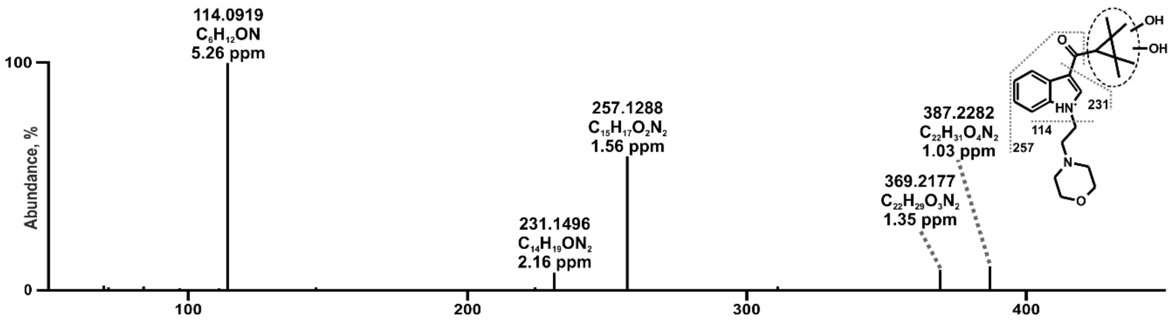

MB21 dihydroxylation at the TMCP part, MS^2 at *m/z* 387.2278, RT 4.31 min

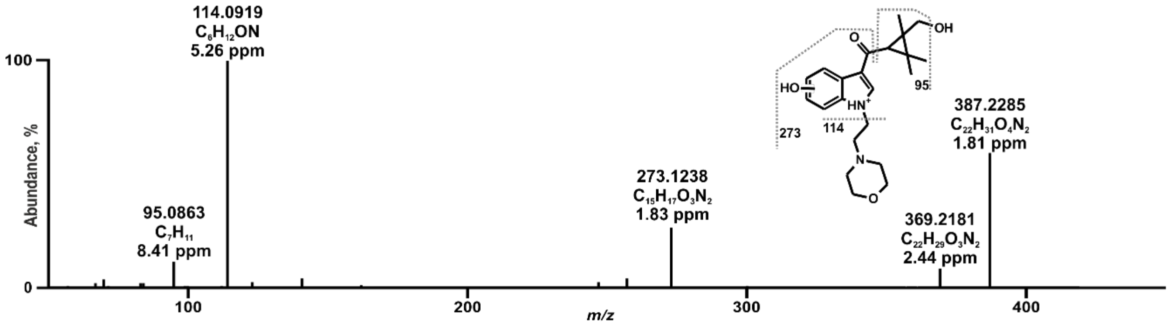

MB22 dihydroxylation at the indole and TMCP, MS^2 at *m/z* 387.2278, RT 4.38 min

Figure S3 continued.

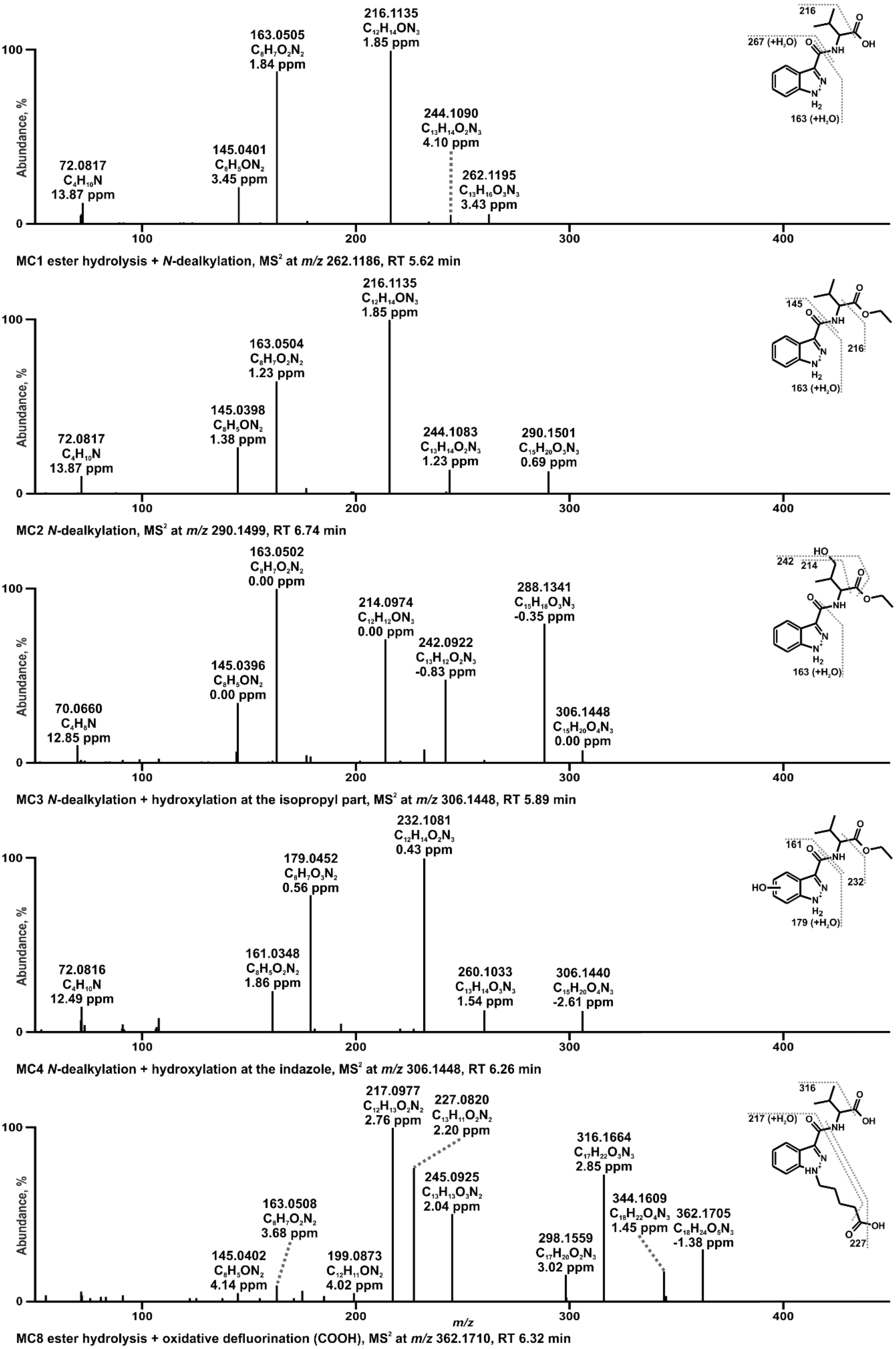

Figure S4 HRMS^2 spectra of the remaining 5F-EMB-PINACA metabolites identified in pooled human liver microsomes or isozyme incubations. Metabolites are ordered by increasing mass and retention time (RT). Metabolite-IDs correspond to Table S3. 5F-EMB-PINACA metabolite (MC).

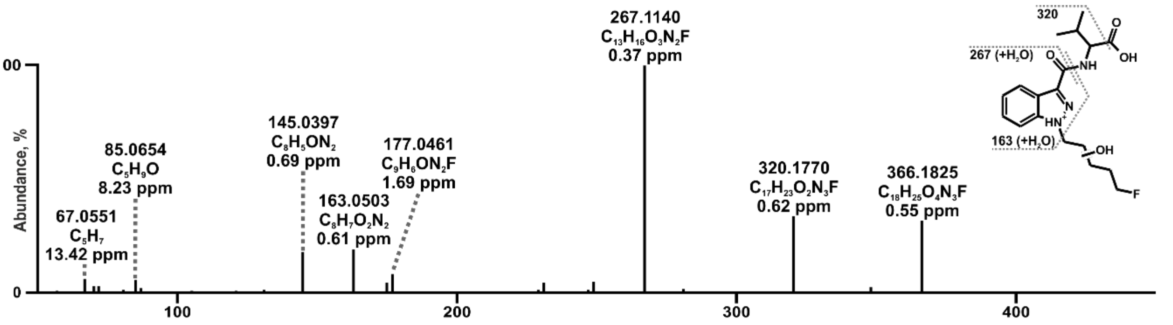

MC9 ester hydrolysis + hydroxylation at the pentyl part, MS^2 at *m/z* 366.1823, RT 6.24 min

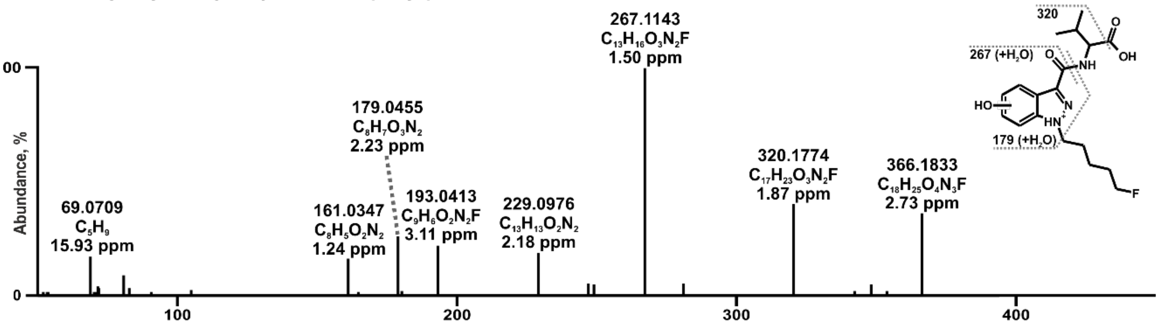

MC11 ester hydrolysis + hydroxylation at the indazole, MS^2 at *m/z* 366.1823, RT 6.89 min

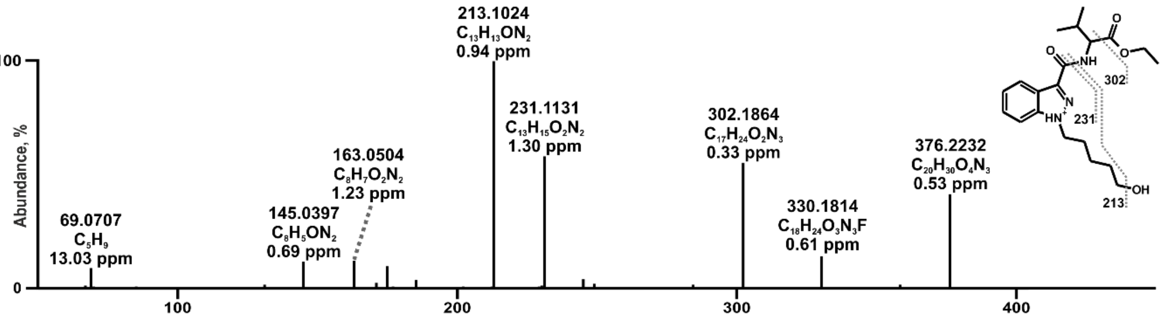

MC12 oxidative defluorination, MS^2 at *m/z* 376.2230, RT 7.33 min

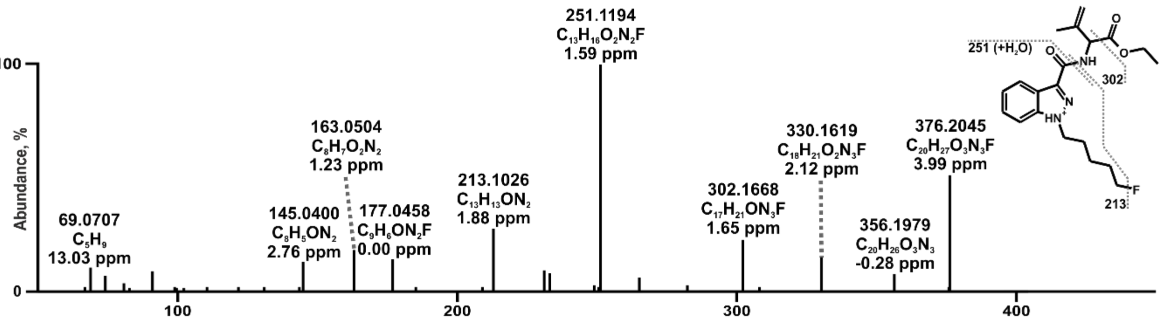

MC13 hydroxylation at the isopropyl part + dehydrogenation, MS^2 at *m/z* 376.2030, RT 8.08 min

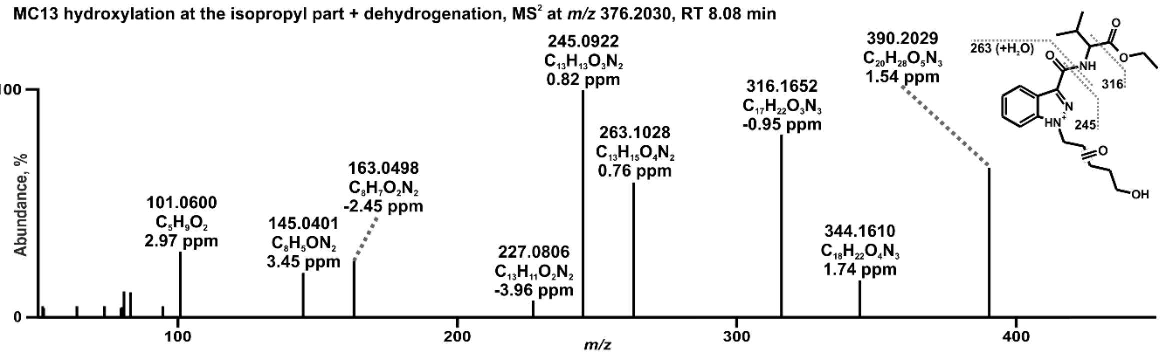

MC14 hydroxylation at the pentyl part + oxidation + oxidative defluorination MS^2 at *m/z* 390.2023, RT 7.06 min

Figure S4 continued.

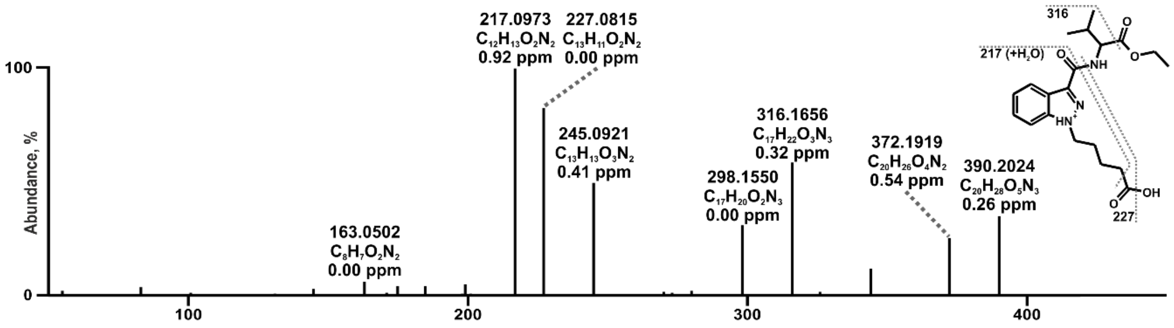

MC15 oxidative defluorination (COOH), MS^2 at *m/z* 390.2023, RT 7.22 min

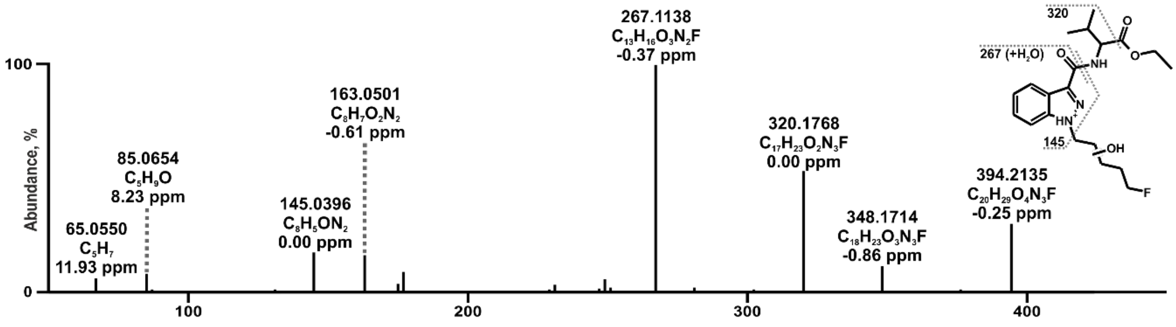

MC16 hydroxylation at the pentyl part, MS^2 at *m/z* 394.2136, RT 7.28 min

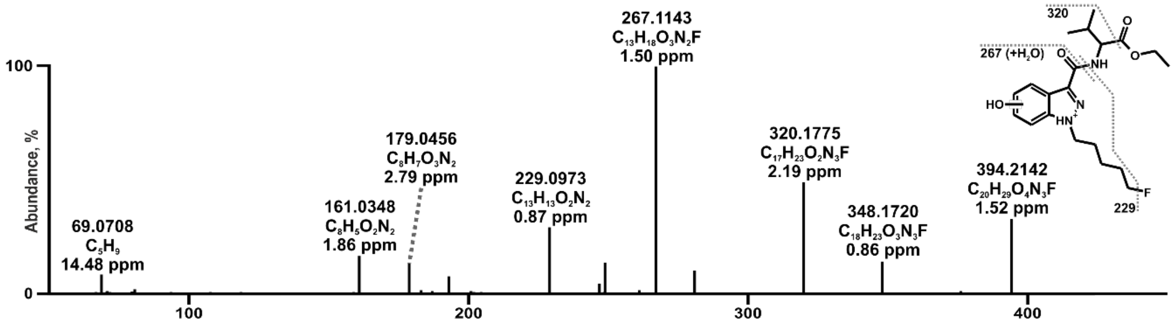

MC17 hydroxylation at the indazole isomer 1, MS^2 at *m/z* 394.2136, RT 7.59 min

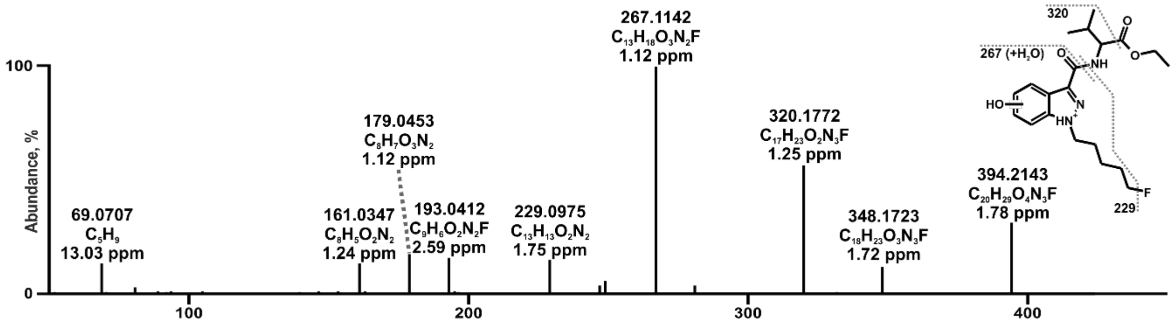

MC18 hydroxylation at the indazole isomer 2, MS^2 at *m/z* 394.2136, RT 7.80 min

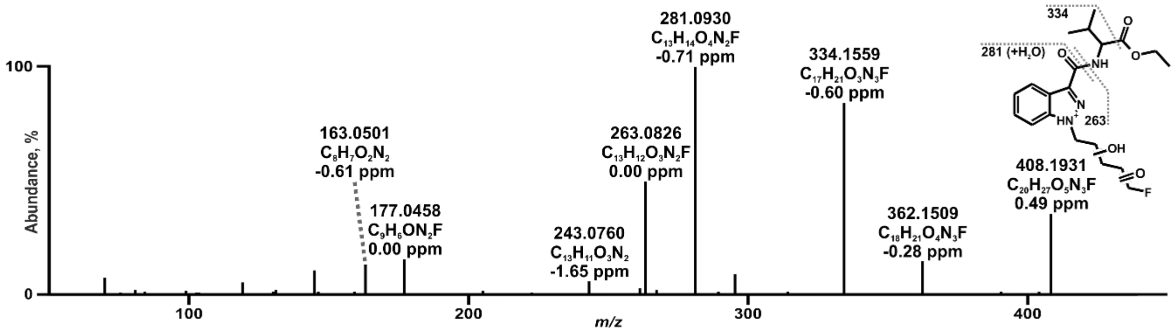

MC19 dihydroxylation at the pentyl part + oxidation, MS^2 at *m/z* 408.1929, RT 7.04 min

Figure S4 continued.

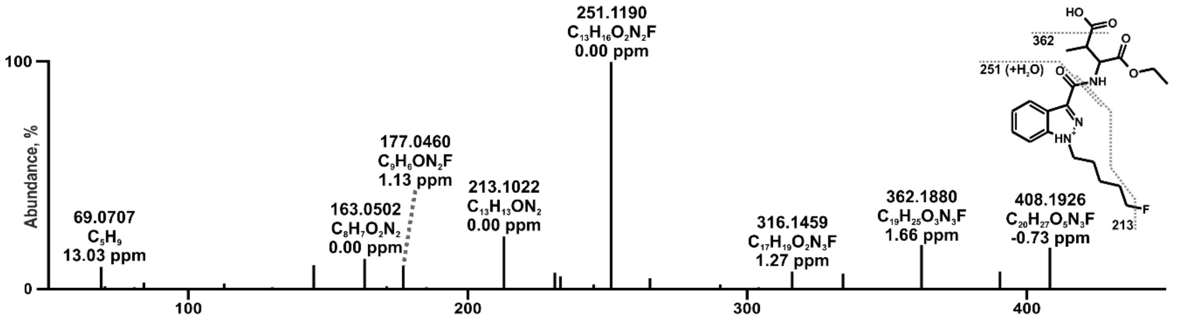

MC20 hydroxylation at the isopropyl part + oxidation (COOH), MS^2 at *m/z* 408.1929, RT 7.29 min

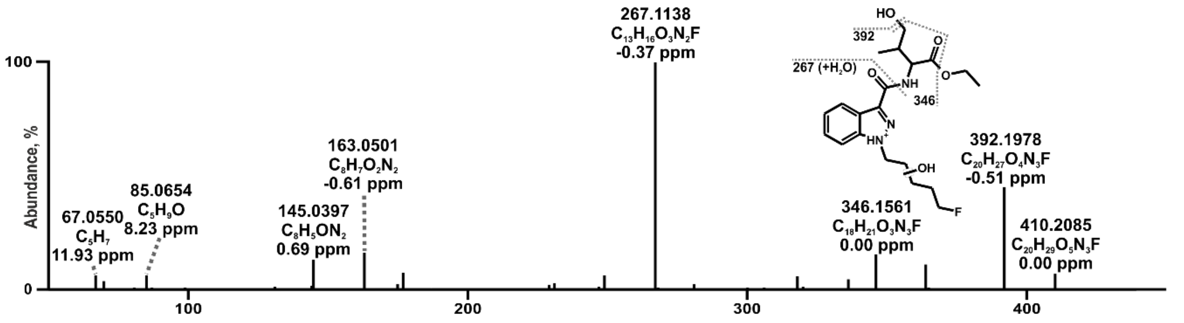

MC21 dihydroxylation at the isopropyl and pentyl part, MS^2 at *m/z* 410.2085, MS^2 at *m/z* 410.2085, RT 6.42 min

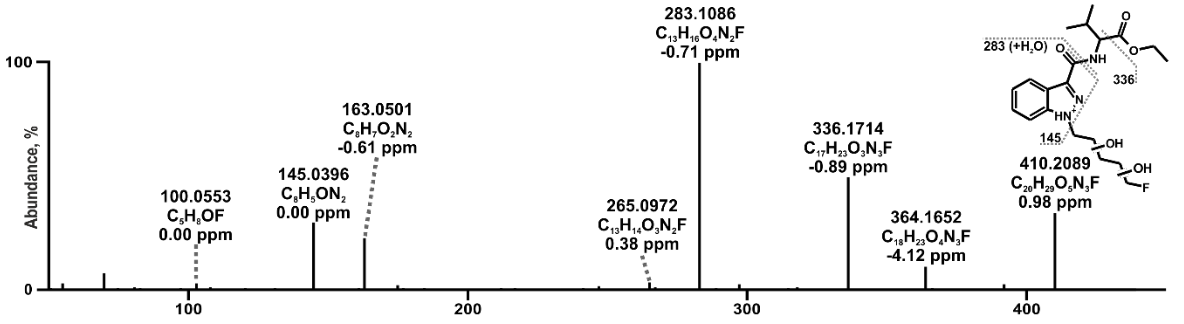

MC22 dihydroxylation at the pentyl part, MS^2 at *m/z* 410.2085, RT 6.68 min

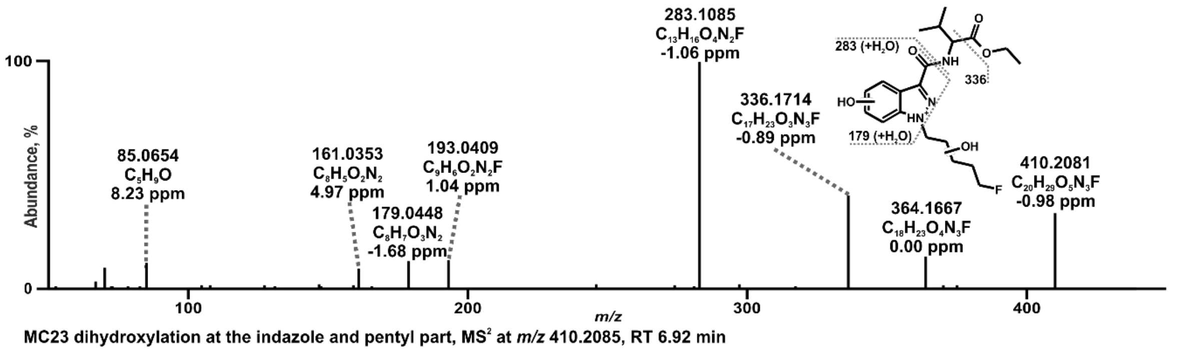

MC23 dihydroxylation at the indazole and pentyl part, MS^2 at *m/z* 410.2085, RT 6.92 min

Figure S4 continued.

Table S1 Detection of JWH-200 and its phase I metabolites in pooled human liver microsomes and isozyme incubations together with their metabolite identification numbers (ID), the calculated exact mass of the protonated molecule (M + H^+), elemental composition, retention time (RT), and the three most abundant fragment ions (FI 1-3) recorded in $HRMS^2$ mode. Metabolites were sorted by increasing mass and RT. Metabolites with an asterisk are regarded as artifacts.

**Metabolite-ID**	**Metabolic reaction**	**Calculated exact mass, *m/z***	**Elemental composition**	**RT, min**	**FI 1, *m/z***	**FI 2, *m/z***	**FI 3, *m/z***
JWH-200	-	385.1910	$C_{25}H_{25}O_2N_2$	6.24	155.0491	114.0913	145.0647
MA1	*N*-Dealkylation	315.1491	$C_{21}H_{19}ON_2$	5.66	155.0491	145.0647	144.0647
MA2	Oxidative morpholine cleavage	316.1332	$C_{21}H_{18}O_2N$	7.08	155.0491	188.0706	145.0647
MA3	Oxidative morpholine cleavage + oxidation (COOH)	330.1124	$C_{21}H_{16}O_3N$	7.02	155.0491	202.0498	145.0647
MA4	*N*-Dealkylation + hydroxylation at the naphthalene	331.1441	$C_{21}H_{19}O_2N_2$	5.05	171.0440	115.0542	274.0862
MA5	Oxidative morpholine cleavage + hydroxylation at the indole isomer 1	332.1281	$C_{21}H_{18}O_3N$	6.20	155.0491	204.0655	145.0647
MA6	Oxidative morpholine cleavage + hydroxylation at the indole isomer 2	332.1281	$C_{21}H_{18}O_3N$	6.34	155.0491	204.0655	145.0647
MA7	Oxidative morpholine cleavage + hydroxylation at the ethyl part	332.1281	$C_{21}H_{18}O_3N$	6.74	155.0491	204.0655	186.0549
MA8	Oxidative morpholine cleavage + epoxidation + non-enzymatic hydrolysis	350.1386	$C_{21}H_{20}O_4N$	5.33	171.0440	188.0706	189.0546
MA9	Oxidative morpholine ring opening isomer 1	359.1754	$C_{23}H_{23}O_2N_2$	5.67	155.0491	88.0756	145.0647
MA10	Oxidative morpholine ring opening + hydroxylation at the naphthalene isomer 1	375.1703	$C_{23}H_{23}O_3N_2$	5.00	171.0440	296.1069	88.0756
MA11	Oxidative morpholine ring opening + hydroxylation at the naphthalene isomer 2	375.1703	$C_{23}H_{23}O_3N_2$	5.08	171.0440	115.0542	88.0756
MA12	Oxidative morpholine ring opening isomer 2	387.1703	$C_{24}H_{23}O_3N_2$	6.66	155.0491	116.0706	145.0647
MA13	Oxidative morpholine ring opening isomer 3	387.1703	$C_{24}H_{23}O_3N_2$	6.75	155.0491	145.0647	116.0706
MA14	Oxidative morpholine opening + epoxidation + non-enzymatic hydrolysis	393.1808	$C_{23}H_{25}O_4N_2$	4.09	171.0440	189.0546	375.1703
MA15	Hydroxylation + oxidation at the ethyl part isomer 1	399.1703	$C_{25}H_{23}O_3N_2$	5.60	155.0491	244.1206	216.0893
MA16	Dihydroxylation at the morpholine + dehydrogenation	399.1703	$C_{25}H_{23}O_3N_2$	6.96	155.0491	128.0706	243.1128

MA17	Hydroxylation + oxidation at the morpholine or ethyl part isomer 2	399.1703	$C_{25}H_{23}O_3N_2$	7.18	155.0491	128.0706	145.0647
MA18	Hydroxylation at the naphthalene isomer 1	401.1859	$C_{25}H_{25}O_3N_2$	5.44	114.0913	171.0440	314.1175
MA19	Hydroxylation at the naphthalene isomer 2	401.1859	$C_{25}H_{25}O_3N_2$	5.53	171.0440	114.0913	115.0542
MA20	Hydroxylation at the naphthalene isomer 3	401.1859	$C_{25}H_{25}O_3N_2$	5.62	114.0913	171.0440	231.1491
MA21*	Hydroxylation at the ethyl part	401.1859	$C_{25}H_{25}O_3N_2$	6.09	298.1226	155.0491	170.0600
MA22	Hydroxylation at the morpholine isomer 1	401.1859	$C_{25}H_{25}O_3N_2$	6.26	155.0491	383.1754	112.0756
MA23	Hydroxylation at the morpholine isomer 2	401.1859	$C_{25}H_{25}O_3N_2$	6.70	155.0491	383.1754	145.0647
MA24	Oxidative morpholine ring opening + hydroxylation at the naphthalene isomer 3	403.1652	$C_{24}H_{23}O_4N_2$	6.23	171.0440	116.0706	161.0597
MA25	Hydroxylation at the morpholine + dehydrogenation + epoxidation + non-enzymatic hydrolysis	417.1808	$C_{25}H_{25}O_4N_2$	5.38	189.0546	171.0440	314.1175
MA26	Dihydroxylation at the morpholine or ethyl part	417.1808	$C_{25}H_{25}O_4N_2$	5.90	155.0491	300.1230	146.0811
MA27	Epoxidation + non-enzymatic hydrolysis isomer 1	419.1965	$C_{25}H_{27}O_4N_2$	4.44	114.0913	171.0440	189.0546
MA28	Epoxidation + non-enzymatic hydrolysis isomer 2	419.1965	$C_{25}H_{27}O_4N_2$	4.86	114.0913	171.0440	401.1859

Table S2 Detection of A-796260 and its phase I metabolites in pooled human liver microsomes and isozyme incubations together with their metabolite identification numbers (ID), the calculated exact mass of the protonated molecule ($M + H^+$), elemental composition, retention time (RT), and the three most abundant fragment ions (FI 1-3) recorded in $HRMS^2$ mode. Metabolites were sorted by increasing mass and RT. Tetramethylcyclopropyl (TMCP). Metabolites with an asterisk are regarded as artifacts.

**Metabolite-ID**	**Metabolic reaction**	**Calculated exact mass, *m/z***	**Elemental composition**	**RT, min**	**FI 1, *m/z***	**FI 2, *m/z***	**FI 3, *m/z***
A-796260	-	355.2380	$C_{22}H_{31}O_2N_2$	6.42	125.0960	114.0913	97.1011
MB1	*N*-Dealkylation	285.1961	$C_{18}H_{25}ON_2$	5.91	125.0960	97.1011	268.1695
MB2	Oxidative morpholine cleavage	286.1801	$C_{18}H_{24}O_2N$	7.36	125.0960	188.0706	268.1695
MB3	*N*-Dealkylation + hydroxylation at the TMCP	301.1910	$C_{18}H_{25}O_2N_2$	4.61	187.0865	144.0443	95.0855
MB4	Oxidative morpholine ring cleavage + hydroxylation at the TMCP	302.1750	$C_{18}H_{24}O_3N$	6.05	188.0706	144.0443	284.1645
MB5	Oxidative morpholine ring cleavage + hydroxylation at the ethyl part	302.1750	$C_{18}H_{24}O_3N$	7.01	125.0960	204.0655	97.1011
MB6	Oxidative morpholine ring opening isomer 1	329.2223	$C_{20}H_{29}O_2N_2$	5.93	125.0960	88.0756	231.1128
MB7	Oxidative morpholine ring opening + hydroxylation at the TMCP isomer 1(hemiketal formation)	345.2172	$C_{20}H_{29}O_3N_2$	4.62	88.0756	231.1128	144.0443
MB8	Hydroxylation at the morpholine + dehydrogenation	353.2223	$C_{22}H_{29}O_2N_2$	7.86	125.0960	255.1128	112.0756
MB9	Oxidative morpholine ring opening isomer 2	357.2172	$C_{21}H_{29}O_3N_2$	6.94	116.0706	125.0960	259.1077
MB10	Oxidative morpholine ring opening isomer 3	357.2172	$C_{21}H_{29}O_3N_2$	7.08	116.0706	125.0960	259.1077
MB11	Dihydroxylation at the TMCP + dehydrogenation	369.2172	$C_{22}H_{29}O_3N_2$	5.38	114.0913	257.1284	231.1491
MB12	Dihydroxylation at the morpholine and ethyl part + dehydrogenation	369.2172	$C_{22}H_{29}O_3N_2$	5.95	125.0960	97.1011	227.0815
MB13	Hydroxylation at the morpholine + oxidation	369.2172	$C_{22}H_{29}O_3N_2$	7.53	128.0706	125.0960	271.1077
MB14	Hydroxylation at the TMCP isomer 1	371.2329	$C_{22}H_{31}O_3N_2$	4.95	114.0913	257.1284	353.2223
MB15	Hydroxylation at the TMCP isomer 2	371.2329	$C_{22}H_{31}O_3N_2$	5.52	114.0913	83.0491	257.1284

MB16	Hydroxylation at the TMCP isomer 3 (hemiketal formation)	371.2329	$C_{22}H_{31}O_3N_2$	5.66	114.0913	287.1390	100.0756
MB17*	Hydroxylation at the ethyl part	371.2329	$C_{22}H_{31}O_3N_2$	6.35	268.1695	125.0960	170.0600
MB18	Hydroxylation at the morpholine or ethyl part	371.2329	$C_{22}H_{31}O_3N_2$	6.42	125.0960	353.2223	112.0756
MB19	Oxidative morpholine ring opening + hydroxylation at the TMCP isomer 2	373.2121	$C_{21}H_{29}O_4N_2$	5.61	116.0706	259.1077	355.2016
MB20	Oxidative morpholine ring opening + hydroxylation at the TMCP isomer 3	373.2121	$C_{21}H_{29}O_4N_2$	5.76	116.0706	259.1077	355.2016
MB21	Dihydroxylation at the TMCP	387.2278	$C_{22}H_{31}O_4N_2$	4.31	114.0913	257.1284	369.2172
MB22	Dihydroxylation at the indole and TMCP	387.2278	$C_{22}H_{31}O_4N_2$	4.38	114.0913	273.1233	95.0855

Table S3 Detection of 5F-EMB-PINACA and its phase I metabolites in pooled human liver microsomes and isozyme incubations together with their metabolite identification numbers (ID), the calculated exact mass of the protonated molecule (M + H$^+$), elemental composition, retention time (RT), and the three most abundant fragment ions (FI 1-3) recorded in HRMS^2 mode. Metabolites were sorted by increasing mass and RT.

**Metabolite-ID**	**Metabolic reaction**	**Calculated exact mass, *m/z***	**Elemental composition**	**RT, min**	**FI 1, *m/z***	**FI 2, *m/z***	**FI 3, *m/z***
5F-EMB-PINACA	-	378.2187	$C_{20}H_{29}O_3N_3F$	8.18	304.1819	251.1190	233.1084
MC1	*N*-Dealkylation + ester hydrolysis	262.1186	$C_{13}H_{16}O_3N_3$	5.62	216.1131	163.0502	145.0396
MC2	*N*-Dealkylation	290.1499	$C_{15}H_{20}O_3N_3$	6.74	216.1131	163.0502	145.0396
MC3	*N*-Dealkylation + hydroxylation at the isopropyl	306.1448	$C_{15}H_{20}O_4N_3$	5.89	163.0502	288.1342	214.0974
MC4	*N*-Dealkylation + hydroxylation at the indazole	306.1448	$C_{15}H_{20}O_4N_3$	6.26	232.1080	179.0451	161.0345
MC5	Ester hydrolysis + oxidative defluorination	348.1917	$C_{18}H_{26}O_4N_3$	6.37	213.1022	231.1128	302.1863
MC6	Ester hydrolysis + lactone formation	348.1717	$C_{18}H_{23}O_3N_3F$	6.91	251.1190	213.1022	163.0502
MC7	Ester hydrolysis	350.1874	$C_{18}H_{25}O_3N_3F$	7.35	251.1190	304.1819	213.1022
MC8	Ester hydrolysis + oxidative defluorination (COOH)	362.1710	$C_{18}H_{24}O_5N_3$	6.32	217.0971	227.0815	316.1655
MC9	Ester hydrolysis + hydroxylation at the pentyl	366.1823	$C_{18}H_{25}O_4N_3F$	6.24	267.1139	320.1768	163.0502
MC10	Ester hydrolysis + hydroxylation at the isopropyl	366.1823	$C_{18}H_{25}O_4N_3F$	6.52	251.1190	348.1717	213.1022
MC11	Ester hydrolysis + hydroxylation at the indazole	366.1823	$C_{18}H_{25}O_4N_3F$	6.89	267.1139	320.1768	179.0451
MC12	Oxidative defluorination	376.2230	$C_{20}H_{30}O_4N_3$	7.33	213.1022	231.1128	302.1863
MC13	Hydroxylation at the isopropyl + dehydrogenation	376.2030	$C_{20}H_{27}O_3N_3F$	8.08	251.1190	213.1022	302.1663
MC14	Hydroxylation at the pentyl + oxidation + oxidative defluorination	390.2023	$C_{20}H_{28}O_5N_3$	7.04	245.0920	316.1655	263.1026
MC15	Oxidative defluorination (COOH)	390.2023	$C_{20}H_{28}O_5N_3$	7.22	217.0971	227.0815	316.1655
MC16	Hydroxylation at the pentyl	394.2136	$C_{20}H_{29}O_4N_3F$	7.28	267.1139	320.1768	145.0396

MC17	Hydroxylation at the indazole isomer 1	394.2136	$C_{20}H_{29}O_4N_3F$	7.59	267.1139	320.1768	229.0971
MC18	Hydroxylation at the indazole isomer 2	394.2136	$C_{20}H_{29}O_4N_3F$	7.80	267.1139	320.1768	179.0451
MC19	Dihydroxylation at the pentyl + oxidation	408.1929	$C_{20}H_{27}O_5N_3F$	7.04	281.0932	334.1561	263.0826
MC20	Hydroxylation at the isopropyl part + oxidation (COOH)	408.1929	$C_{20}H_{27}O_5N_3F$	7.29	251.1190	213.1022	362.1874
MC21	Dihydroxylation at the isopropyl and pentyl	410.2085	$C_{20}H_{29}O_5N_3F$	6.42	267.1139	392.1980	163.0502
MC22	Dihydroxylation at the pentyl	410.2085	$C_{20}H_{29}O_5N_3F$	6.68	283.1088	336.1717	145.0396
MC23	Dihydroxylation at the indazole and pentyl	410.2085	$C_{20}H_{29}O_5N_3F$	6.92	283.1088	336.1717	364.1667

Table S4 Isozyme mapping of initial JWH-200 metabolites in comparison to pooled human liver microsomes (pHLM) and flavin-containing monooxygenase (FMO) 3 incubations. Metabolite-IDs correspond to Table S1. Cytochrome P450 (CYP); +, detected; - not detected.

**Enzyme incubations**	**Initial metabolic reactions and metabolite-IDs**				
	N-Dealkylation (MA1)	Oxidative morpholine cleavage (MA2)	Oxidative morpholine ring opening (MA9, MA12, MA13)	Hydroxylation (MA18-MA23)	Dihydrodiol formation (MA27, MA28)
CYP1A2	+	-	+	+	+
CYP2A6	-	-	-	-	-
CYP2B6	-	-	+	-	-
CYP2C8	-	-	+	+	-
CYP2C9	-	-	-	+	-
CYP2C19	-	-	+	+	+
CYP2D6	-	-	+	+	-
CYP2E1	-	-	-	-	-
CYP3A4	+	+	+	+	+
CYP3A5	+	+	+	+	+
FMO3	-	-	-	-	-
pHLM	+	+	+	+	+

Table S5 Isozyme mapping of initial A-796260 metabolites in comparison to pooled human liver microsomes (pHLM) and flavin-containing monooxygenase (FMO) 3 incubations. Metabolite-IDs correspond to Table S2. Cytochrome P450 (CYP); +, detected; - not detected.

**Enzyme incubations**	**Initial metabolic reactions and metabolite-IDs**			
	N-Dealkylation (MB1)	Oxidative morpholine cleavage (MB2)	Oxidative morpholine ring opening (MB6, MB9, MB10)	Hydroxylation (MB14-MB18)
CYP1A2	+	-	+	+
CYP2A6	-	-	-	-
CYP2B6	-	-	-	-
CYP2C8	-	-	+	+
CYP2C9	-	-	-	+
CYP2C19	-	-	+	-
CYP2D6	-	-	+	+
CYP2E1	-	-	-	-
CYP3A4	+	+	+	+
CYP3A5	+	+	+	+
FMO3	-	-	-	-
pHLM	+	+	+	+

Table S6 Isozyme mapping of initial 5F-EMB-PINACA metabolites in comparison to pooled human liver microsomes (pHLM) and flavin-containing monooxygenase (FMO) 3 incubations. Metabolite IDs correspond to Table S3. Cytochrome P450 (CYP); +, detected; - not detected.

**Enzyme incubations**	**Initial metabolic reactions and metabolite-IDs**			
	N-Dealkylation (MC2)	Ester hydrolysis (MC7)	Oxidative defluorination (MC12)	Hydroxylation (MC16-MC18)
CYP1A2	+	-	+	+
CYP2A6	-	-	-	-
CYP2B6	+	-	+	+
CYP2C8	-	-	+	+
CYP2C9	-	-	+	+
CYP2C19	+	-	+	+
CYP2D6	-	-	+	-
CYP2E1	-	-	-	-
CYP3A4	+	-	-	+
CYP3A5	+	-	+	+
FMO3	-	-	-	-
pHLM	-	+	-	-
